# Enhancing therapeutic antibody profiling: orthogonal strategies for stability and quality assessment

**DOI:** 10.3389/fphar.2025.1667210

**Published:** 2025-09-26

**Authors:** Nicole G. Metzendorf, Inga Petersen, Greta Hultqvist

**Affiliations:** Department of Pharmacy, Uppsala University, Uppsala, Sweden

**Keywords:** antibody engineering forum, biophysical characterisation, conformational stability, aggregation propensity, therapeutic developement

## Abstract

Today, a variety of multivalent antibody formats can be engineered, offering significant flexibility for therapeutic and diagnostic approaches. While unmodified antibodies have evolved to be structurally stable, extensive engineering, such as domain fusion or size reduction, can compromise their thermal stability, conformational integrity, and overall functional performance. As a result, reliable antibody development requires rigorous biophysical characterization to ensure protein quality, including assessments of purity, folding, stability and aggregation propensity. In this study, we systematically evaluate a panel of analytical methods, including SDS-PAGE, nano differential scanning fluorimetry (nanoDSF), dynamic light scattering (DLS), size exclusion chromatography (SEC), mass photometry, circular dichroism (CD), small-angle X-ray scattering (SAXS) and electron microscopy, to characterize a series of antibody-derived constructs. These include a full-length IgG (Ab1), a bivalent fusion antibody (Ab1-scFv1), a bispecific tandem single-chain fragment variable (bi-scFv2-scFv1) and single-chain variable fragments (scFv1, scFv3, and scFv4). These constructs served as representative model proteins to assess method performance and sensitivity to structural and biophysical differences. Our results show that full-length antibodies (Ab1 and Ab1-scFv1) exhibit high thermal and structural stability and remain predominantly monomeric across all tested conditions. In contrast, engineered fragments, particularly bi-scFv2-scFv1 and scFv variants, display increased aggregation propensity and reduced conformational stability, as evidenced by higher polydispersity in DLS, early elution peaks in SEC, and altered thermal folding profiles in nanoDSF. SAXS and CD further revealed extended, flexible conformations in larger constructs and partial folding deficiencies in smaller fragments. Overall, this study underscores the importance of integrating orthogonal analytical methods to ensure a robust evaluation of antibody format stability and integrity. With the increasing complexity of engineered antibody therapeutics, these tools offer practical insights into selecting appropriate constructs for downstream development, enhancing experimental reproducibility, and mitigating risk in early-stage research and therapeutic design. Furthermore, many of the assessed quality attributes, such as monodispersity, conformational stability, and aggregation behaviour, are directly relevant to *in vivo* performances, including pharmacokinetics and immunogenicity making such characterization essential for advancing antibody candidates toward clinical applications.

## 1 Introduction

The field of protein biotherapeutics is rapidly expanding, attracting a growing number of new scientists. While proteins are highly functional and versatile molecules, their structural complexity and sensitivity to environmental conditions present significant challenges, particularly in therapeutic applications (Kumar et al., 2024). Ensuring consistency, safety, and efficacy requires rigorous quality control and analytical measures. This is especially critical in the development of therapeutic antibodies and related products, which are commonly produced in mammalian cell culture systems. These products exhibit unique structural and functional characteristics, such as glycosylation patterns, a propensity for aggregation, and potential immunogenicity. Initial *in vitro* experiments and preclinical *in vivo* analyses of transient proteins must be conducted efficiently and effectively prior to large-scale production. Therefore, comprehensive analytical characterisation and stringent quality control are essential. In this manuscript, we present a selection of recommended methods for assessing protein quality prior to conducting *in vitro* and *in vivo* assays. We also highlight additionally optional techniques for further characterization of the recombinant proteins.

In the context of protein-based therapeutics, antibodies, particularly the standard IgG format, are widely used and frequently engineered to enhance functionality (Damelang et al., 2024). A common modification is a single-chain variable fragment (scFv), the smallest antibody unit that retains antigen-binding ability (Boado et al., 2009). It consists of a variable heavy chain (VH) linked to the variable light chain (VL) connected by a flexible peptide linker. scFv’s can be integrated into antibody constructs to confer dual specificity, with flexible positioning within the IgG format and the potential for multiple scFv insertions (Ahamadi-Fesharaki et al., 2019; Gezehagn Kussia and Tessema, 2024). In previous work we have developed bispecific antibodies (Hultqvist et al., 2017) as well as hexavalent-antibody constructs (Petersen et al., 2023; Rofo et al., 2021), and explored engineered Fc fragments such as single-chain Fc domains (scFc) (Morrison et al., 2023; Levin et al., 2015). In this study, we selected a full-length antibody (Ab1), a bivalent construct (Ab1-scFv1), a bispecific tandem scFv (bi-scFv2-scFv1) and three individual scFv’s (scFv1, scFv3, and scFv4) to evaluate various analytical methods before downstream application.

In scFv’s, the limited interface between variable heavy and light chains can promote unintended intermolecular interactions. Instead of folding intramolecularly, some scFv’s form multimers by pairing with domains from other molecules, altering their binding properties (Hudson and Kortt, 1999; Arslan et al., 2022). Multimerization refers to the specific, often reversible assembly of protein subunits into defined oligomers, while aggregation typically involves non-specific, irreversible clumping into larger, often dysfunctional complexes (Jahn and Radford, 2008). Similarly, Fc domains can drive dimerization or higher-order oligomerization through disulfide bonds or non-covalent interactions, especially in Fc fusion proteins, under non-reducing conditions or when the host cell folding machinery is stressed (Wu et al., 2001; Strand et al., 2013; Lowe et al., 2011). Multimerization is not exclusive to scFv’s or Fc-fusion proteins, other recombinant proteins and antibody fragments can also oligomerize via domain swapping, disulfide bridges, or hydrophobic interactions (Bennett et al., 1995; Wallis and Drickamer, 1999). Protein aggregation or multimerization reduces monomer levels and may cause adverse effects in in vivo applications (Jahn and Radford, 2008). Antibody complexes can enhance immune responses, potentially triggering inflammation and anti-drug antibodies (ADA) that compromise therapeutic efficiency and safety (Ratanji et al., 2014; Lundahl et al., 2021; Rahban et al., 2023).

In this study, we compare a set of widely used biophysical and biochemical methods for the characterization of antibody-based therapeutic candidates. Rather than proposing a fixed analytical workflow, our aim is to highlight the strengths, limitations, and complementarity of commonly available methods and encourage the combined use of orthogonal techniques for a more reliable and reproducible quality assessment. This side-by-side comparison fills a gap in literature, where analytical methods are often described individually but rarely evaluated comparatively on therapeutically relevant constructs. The study is intended as a practical reference to build robust characterization strategies based on construct format and downstream application.

**FIGURE 1:**
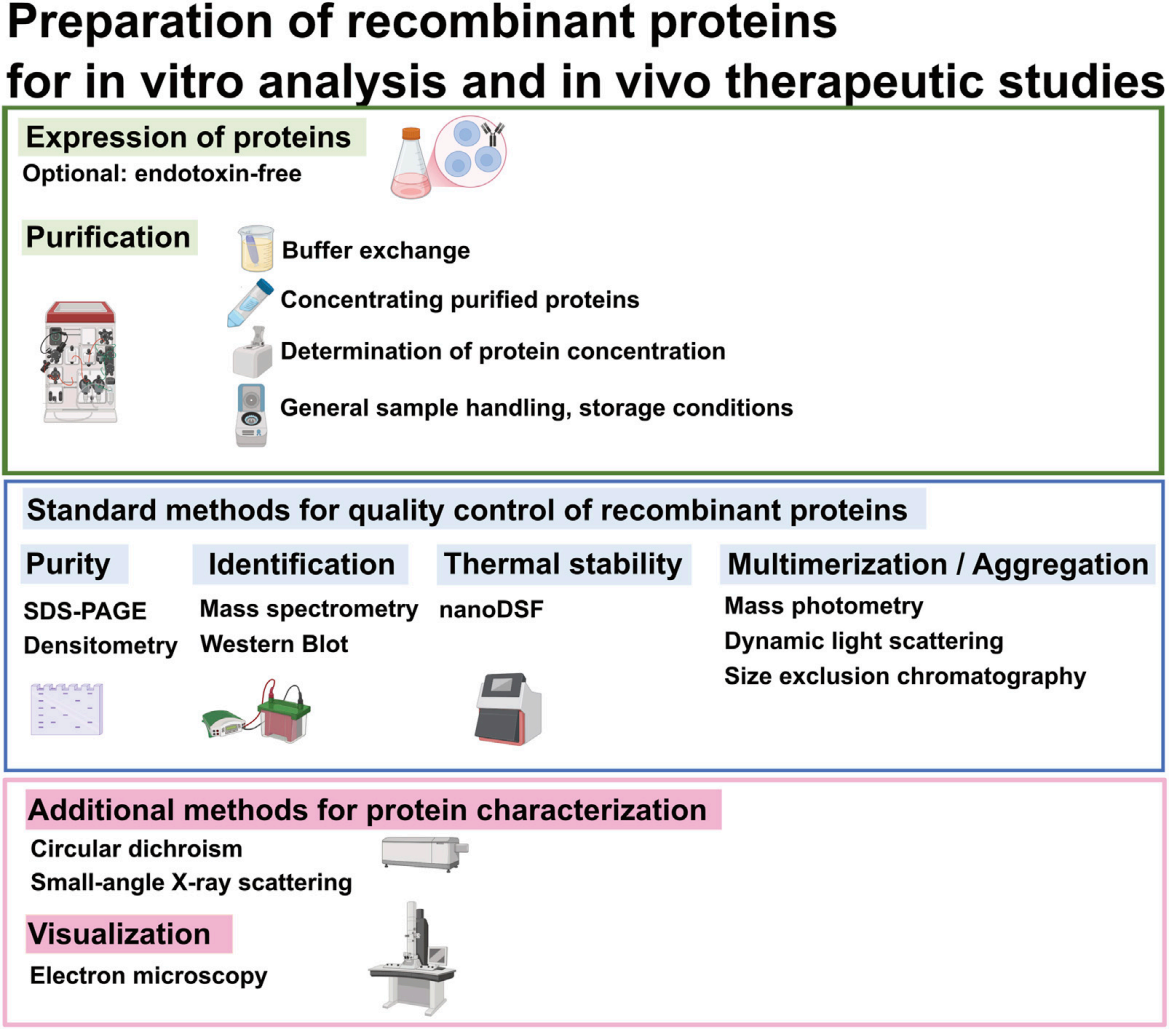
Flowchart of analytical protein preparation for *in vitro* and *in vivo* assays. This diagram outlines standard methods for quality control of recombinant proteins, along with optional techniques for further protein characterization and visualization, as needed.

Proper handling of recombinant proteins after purification is essential to preserve protein integrity and function. Key considerations include storage conditions and buffer selection to preserve protein stability and prevent protein aggregation. This paper summarizes and compares analytical methods for assessing structural and biophysical quality attributes relevant to therapeutic antibody development, including purity (e.g., SDS-PAGE, densitometry), identity (e.g., mass spectrometry, Western blot), and stability (e.g., nano Differential Scanning Fluorimetry (nanoDSF)). We also include techniques to evaluate multimerization and aggregation (mass photometry, dynamic light scattering (DLS), and size exclusion chromatography (SEC)), along with optional tools for conformational analysis and visualization such as circular dichroism (CD), small-angle X-ray scattering (SAXS), negative stain electron microscopy (EM) (Figure 1). Each method is evaluated based on practical considerations such as sensitivity, throughput and suitability for early-stage construct screening.

## 2 Materials and equipment

List of required equipment:

ÄKTA Start system (Cytiva)

Micro spectrophotometer (Nanodrop)

SDS PAGE and Western blot equipment

Odyssey Fc Machine

Prometheus Panta NT48 instrument (NanoTemper Technologies GmbH)

JASCO J-1500 CD spectrometer (JASCO)

Superdex Increase 10/300 (Cytiva)

Anton Paar Litesizer 100 (Anton Paar GmbH)

Refeyn 2 MP (Refeyn Ltd.)

SAXS at beamline B21, Diamond Light Source, United Kingdom

Tecnai™ G2 Spirit BioTwin transmission electron microscope (Thermo Fisher/FEI)

## 3 Materials and methods

### 3.1 Expression and purification of recombinant proteins

The recombinant proteins Ab1, Ab1-scFv1, bi-scFv2-scFv1, scFv1, scFv3, and scFv4 were expressed in Expi293 cells (cat. no. A14527, Thermofisher) and purified by using ÄKTA Start system (Cytiva) and Protein-G columns (cat. no. 17-0405-01, Cytiva) as described previously (Hultqvist et al., 2017; Fang et al., 2017). Briefly, Expi293 cells were transiently transfected with pcDNA3.4 vectors using polyethyleneimine (PEI, cat. no. A14527, Polyscience) as the transfection reagent. The culture supernatant was harvested after 6 days post-transfection, clarified using Cellpure (cat. no. 525243, Sigma-Aldrich), and filtered through a 0.2 µM polyether sulfone (PES) membrane (cat. no. GWP04700, Millipore). The clarified supernatant was then applied to a Protein-G column (cat. no. GE17-0405-03, Cytiva) for purification. Bound proteins were eluted with 0.7% acetic acid (cat. no. 33209, Sigma-Aldrich), followed by buffer exchange to PBS (cat. no. 14190250, Thermofisher) using 7K desalting columns (cat. no. 89892, Thermo Scientific). Protein concentrations were determined by absorbance at 280 nm (A280) by using a micro spectrophotometer (Nanodrop 200C, Thermo Scientific) and the theoretical extinction coefficient (in M^−1^*cm^−1^) of the recombinant proteins were calculated from the amino acid sequences via the ExPASy ProtParam tool.

### 3.2 SDS-PAGE and Western blot

Purified recombinant proteins were mixed with 25% LDS sample buffer (cat. no. B0007, Life Technologies) with or without reducing agent (DTT, 1xBolt sample reducing agent, cat. no. B0004, Life technologies), loaded onto a 4%–12% Bis-Tris protein gel (cat. no. NW04125BOX, Invitrogen) and run at 80 V for analysis. The gel was stained with PAGE blue protein staining solution (cat. no. 24620, Thermo Scientific). Molecular size was determined by comparing the achieved bands to a pre-stained protein ladder (PageRulerTM Plus Pre-stained Protein Ladder, 10–250 kDa (cat. no. 26619, Thermo Scientific). Images were obtained by using the Odyssey Fc Machine (Li-COR Biosciences). For densitometric analysis of protein purity, band intensities were analysed using ImageJ software (ImageJ 1.51m9) by measuring the relative density of each band. Background was subtracted from all lanes prior to quantification to ensure accurate analysis of band intensities.

Western blot transfer was performed for 2 h at 100 V onto PVDF membranes (cat. no. 88520, Life Technologies). Membranes were blocked with 5% non-fat dry milk in TBS-Tween for 1 h at RT. For detection of His-tagged proteins, membranes were incubated with HRP-conjugated anti-His antibody (cat. no. HRP-66005, ThermoFisher Scientific). For Fc-containing proteins, membranes were incubated with HRP-conjugated goat-anti-mouse IgG antibody (cat. no. 12-349, Merck). Antibody incubations were carried out for 1 h at RT with gentle agitation. Following incubation, membranes were washed with TBS-Tween buffer and developed using Novex ECL chemiluminescent substrate (cat. no. 10348463, Fisher Scientific). Imaging was performed using the Li-COR odyssey Fc machine (Li-COR Biosciences).

### 3.3 Structural and thermal stability assessment (nanoDSF)

The structural and thermal stability of the recombinant proteins was evaluated by using the Prometheus Panta NT48 instrument (NanoTemper Technologies GmbH, Munich, Germany) and measuring the nano differential scanning fluorimetry (nanoDSF) (Wen et al., 2020). Briefly, equimolar concentrations (2 μM) of the proteins were loaded into glass capillaries (NanoTemper Technologies GmbH, Munich, Germany) and subjected to a linear temperature gradient from 30 °C to 95 °C. Intrinsic tryptophan fluorescence was recorded at 330 nm and 350 nm, and the ratio of fluorescence intensities (350 nm/330 nm) was calculated. The first derivative of this ratio was used to identify the inflection temperature (Ti), which represents major unfolding events and provide a measure of thermal stability. Protein thermal stability was also tested under different buffer conditions such as PBS, 1:5 (v/v) diluted PBS, and water.

### 3.4 Circular dichroism (CD)

CD spectra were recorded between 190 and 260 nm using a JASCO J-1500 CD spectrometer (JASCO, Easton, MD, United States) at 25 °C. Thermal unfolding (Tmelt) measurements were performed by applying a temperature gradient from 4 °C-95 °C. All measurements were performed in 1 mm path length quartz cuvettes (cat. no. 110-1-40, Hellma Analytics) using samples diluted to a final concentration of 2 µM in PBS. Spectra were acquired at a scanning speed of 50 nm/min with a step size of 0.1 nm. For each sample, baseline correction was applied by subtracting the buffer spectrum.

### 3.5 Size exclusion chromatography (SEC)

Size exclusion chromatography was performed using a Superdex Increase 10/300 (Cytiva), which was equilibrated with at least three column volumes PBS (cat. no. 14190250, Thermofisher). For each run, 50 µg of purified protein was diluted in PBS to a maximum volume of 250 µL and loaded onto the column via a 500 µL injection loop. The sample was eluted with PBS at a flow rate of 0.5 mL/min. Eluted fractions were collected every 0.5 mL across the entire elution range (24 mL) and analysed by SDS-PAGE, followed by Coomassie blue staining or Western blot analysis, depending on the downstream analysis. For molecular weight calibration, a high molecular weight SEC standard mix was used under identical conditions. The standard contained the following proteins: Thyroglobulin 669 kDa, Ferritin 440 kDa, Aldolase 158 kDa, Conalbumin 75 kDa, Ovalbumin 43 kDa, Carbonic anhydrase 29 kDa. The elution volume for each protein was used to generate a calibration curve, allowing estimation of the apparent molecular weight of the eluted sample. SEC experiments were performed at RT.

### 3.6 Dynamic light scattering (DLS)

To further assess the quality of the purified proteins, dynamic light scattering (DLS) measurements of the recombinant proteins (2 µM) was performed on an Anton Paar Litesizer 100 (Anton Paar GmbH, Graz, Austria). The size distribution was analysed based on intensity-weighted, on volume-weighted and on number-weighted models. The size of the hydrodynamic diameter was determined based on the intensity-weighted model. Bovine serum albumin (BSA) monomer (cat. no. 421501J, VWR) in PBS was used as a reference standard for monomodal distribution in particle diameter measurements. The polydispersity index (PDI) was calculated to evaluate the uniformity of the particle size distribution. The effects of protein concentration (0.125, 0.25, 0.5, and 1 mg/mL), freeze-thaw cycles, and sample centrifugation on protein stability were also evaluated.

### 3.7 Mass photometry

The purity of the recombinant proteins was validated using mass photometry, performed on a Refeyn 2 MP (Refeyn Ltd., Oxford, United Kingdom). This technique determines molecular mass on the proportional relationship between the intensity of light scattering generated by the molecules interacting with the glass surface and their molecular mass (Young et al., 2018). The data is presented as histograms of mass distribution. Values below zero in the histograms correspond to buffer impurities, which were considered negligible for the proteins measured in this experiment. A commercial purchased IgG was used as a calibrant to determine the molecular weight of the tested proteins.

### 3.8 Small-angle X-ray scattering (SAXS)

SAXS measurements were performed at beamline B21, Diamond Light Source, United Kingdom (proposal 23773). For batch experiments, 25 µL of each samples was measured at four different concentrations: 0.125, 0.25, 0.5, and 1 mg/mL in PBS pH 7.5 were analysed. All samples were centrifuged et 15,000 x g for 10 min at 4 °C before measurement to minimize aggregation. Data were recorded on an Eiger 4M detector with a fixed camera length of 4.014 m and 12.4 keV energy, allowing an angular q range of 0.0038–0.42 Å^-1^. For each concentration, 20 sequential measurements were collected and manually inspected for radiation damage. Identical PBS buffer was measured before and after each sample, and buffer subtraction was carried out using PRIMUS. Only frames without visible radiation damage were averaged.

Radius of gyration (Rg) was determined via Guinier analysis (q Rg < 1.3 criterion) using BioXTAS RAW, and maximum particle dimension (D_max_), and pair distribution function (P(r)) were computed using GNOM. Molecular weights were estimated via volume-of-correlation and extrapolated (I(0)), and compared against theoretical values based on the primary sequence using RAW. Shape reconstructions (bead models) were generated usingIFT GNOM and IFT BIFT, with ten independent models averaged and aligned using DAMAVER and saved as DAMMIF/N files. The filtered model were visualized and further adapted in PyMOL (TM, version 2.6.0, Schrodinger LLC). For comparison with known structures, SAXS envelopes were aligned to the AlphaFold 2-predicted structure of scFv1 and the PDB structure of IgG2a (PBD ID: 1igt).

### 3.9 Electron transmission microscopy - negative staining

A 5 µL drop of the sample was placed on a formvar- and carbon coated 200-mesh copper grid (Ted Pella). The excess solution was removed by blotting with filter paper. The sample was then directly contrasted with 2% uranyl acetate. Eexcess of uranyl acetate was removed by blotting on filter paper. Images were acquired by a Tecnai™ G2 Spirit BioTwin transmission electron microscope (Thermo Fisher/FEI) at 80 kV with an ORIUS SC200 CCD camera and Gatan Digital Micrograph software (both from Gatan Inc./Blue Scientific). Micrographs were analysed using ImageJ software (ImageJ 1.51m9), and the area of the antibodies (n = 40) were measured. The data were quantified and visualized as graph with standard deviation using GraphPad Prism (version 10.4.2 build 534).

## 4 Results

### 4.1 Expression of recombinant antibody constructs

#### 4.1.1 Design of recombinant proteins

**FIGURE 2:**
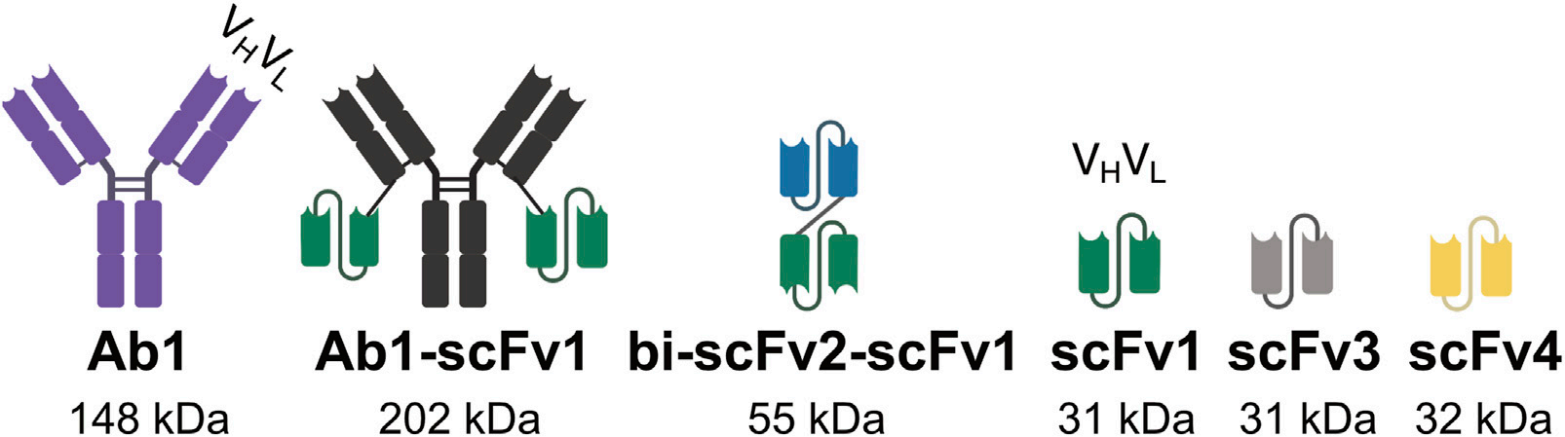
Design of antibodies, multivalent antibodies, and antibody fragments used in this study. Schematic representation of the molecular designs of the antibody constructs evaluated in this study, including full‐length antibodies, engineered multivalent formats, and antibody fragments. The illustrations highlight structural features relevant to binding valency, molecular configuration, and domain composition.

A panel of recombinantly expressed antibody-based constructs, which are routinely used in our laboratory, were selected as model proteins for this study (Figure 2). These include a full-length IgG antibody (Ab1), a bivalent antibody (Ab1-scFv1), a bispecific tandem single-chain fragment variable (bi-scFv2-scFv1) and several single-chain fragment variables (scFv1, scFv3, and scFv4). The following constructs bi-scFv2-scFv1, scFv1, scFv3, and scFv4 were engineered with a C-terminal His-tag to facilitate purification via affinity chromatography.

Expression was carried out in Expi293 cells, a suspension adapted derivative of human embryonic kidney (HEK) 293 cells optimized for high-yield, transient protein expression (Fang et al., 2017). This system was selected for its ability to support complex protein folding and human-like post-translational modifications, which are critical for antibody functionality. Transient transfection offered a rapid and scalable platform for producing multiple constructs under consistent conditions. Constructs were not expressed in bacterial systems, which lack the necessary machinery for proper glycosylation and often result in misfolded or inactive antibody fragments (Caltag Medsystems, 2022). This experimental setup allowed us to directly compare expression levels and yields across different antibody formats, establishing a foundation for downstream purification and functional analysis.

Recombinant expression in Expi293 cells resulted in variable protein yields depending on the format of the antibody construct. Ab1 and Ab1-scFv1 were consistently expressed at high levels, with typical yields of approximately 4–5 mg/L of culture supernatant. In contrast, the bispecific tandem scFv construct bi-scFv2-scFv1, which incorporates two scFv domains in a single polypeptide chain, yielded significantly less protein, ranging from 1–2 mg/L culture supernatant.

Single-chain fragment variables (scFv1, scFv3, and scFv4) showed intermediate expression levels, typically yielding 2–3 mg/L of culture supernatant. These differences likely reflect variations in protein folding efficiency, stability, or expression kinetics inherent to each format. These quantitative benchmarks serve as a reference for evaluating subsequent purification efficiency and functional activity across antibody designs.

### 4.2 Purification

Recombinant protein constructs were purified using affinity chromatography strategies tailored to their structural features. Constructs containing an Fc region, such as Ab1 and Ab1-scFv1 were purified using protein G chromatography, which exploits the high-affinity between protein G and the Fc domain. In contrast, constructs lacking the Fc region, including bi-scFv2-scFv1, scFv1, scFv3, and scFv4, were purified using immobilized metal affinity chromatography (IMAC) via their engineered C-terminal His-tag. For most constructs, a single purification step was sufficient to obtain protein preparations of adequate purity for preclinical research applications. However, in certain cases, additional purification steps, such as ion exchange chromatography, were necessary to improve purity or remove low-level contaminants. The need for further polishing varied depending on the specific construct.

Post-purification, standard protein handling steps were implemented, including buffer exchange, protein concentration, quantification by UV absorbance. These procedures ensured consistency across samples and prepared the proteins for downstream applications.

#### 4.2.1 Buffer exchange and sample preparation

Following affinity purification, buffer exchange was performed to prepare protein samples for downstream assays and improve storage stability. Depending on sample volume and experimental requirements, two size-based buffer exchange methods were routinely employed: dialysis and pre-packed desalting columns.

For larger volumes or particularly sensitive constructs, dialysis provided a gentle and effective method for removing salts and other small molecules. Multiple buffer changes are recommended to ensure complete buffer exchange. The volume of the dialysis buffer should be at least 100-times the sample volume to maintain efficient exchange, but 200–500-times is often recommended. During the initial hours of dialysis, buffer exchange occurs most rapidly. To optimize this process, it is recommended to change the buffer every 2–3 h, followed by an additional overnight exchange.

Smaller sample volumes were typically processed using pre-packed size-exclusion columns, allowing rapid buffer exchange with minimal sample loss. Both approaches rely on molecular weight-based separation, ensuring retention of the target protein while permitting the passage of small molecules.

Buffer exchange proved essential for achieving consistent sample quality and compatibility with downstream analysis. The choice of method was guided by construct-specific factors such as yield, stability, and intended use, as well as practical considerations like sample volume. Details on buffer selection and its impact on performance are discussed in the following chapter.

#### 4.2.2 Buffer selection and protein stability

Buffer composition can significantly influence protein stability, particularly for constructs prone to aggregation and degradation. Protein aggregation occurs often at low pH, during affinity chromatography, were low pH buffers are used to elute bound proteins. Neutralisation is then required to prevent aggregation and preserve protein stability (Pang et al., 2023; Joshi et al., 2014).

To assess the impact of buffer conditions on thermal stability, we compared Ab1 and Ab1-scFv1 in phosphate-buffered saline (PBS), 1:5 diluted PBS and water. As shown in Supplementary Figure S1, both constructs exhibited similar thermal stability profiles across all tested conditions, indicating minimal sensitivity to ionic strength or buffer composition. These results suggest that Ab1 and Ab1-scFv1 are relatively stable proteins, tolerating a range of buffer environments without substantial loss of thermal stability. Based on these findings and considering the requirements for *in vivo* applications, all proteins in this study were stored in PBS, which offers physiological compatibility and minimizes adverse effects during animal experiments.

Although more fragile proteins may require careful buffer optimization based on properties such as isoelectric point (pI) and pH sensitivity, the constructs evaluated here did not show evidence of aggregation or instability under the tested conditions (Rahban et al., 2023).

#### 4.2.3 Protein concentration and aggregation risk

Protein concentration is a critical factor influencing aggregation, particularly during early handling of new constructs. To minimize risk, we generally avoid concentrating proteins, unless higher concentrations are required for downstream applications (Pang et al., 2023; Fields et al., 1992; Knowles et al., 2014; Dobson et al., 1998). When necessary, concentrations are selected based on protein format and prior experience: approximately 33 µM (5 mg/mL) for monoclonal antibodies, approximately 5 µM (1 mg/mL) for multivalent formats, and equivalent molar concentrations for smaller fragments.

To assess the effect of protein concentration on biophysical behaviour, we analysed the bivalent construct Ab1-scFv1 at four concentrations (1, 0.5, 0.25, and 0.125 mg/mL) using DLS and nanoDSF. As shown in Supplementary Figure S2, no significant changes were observed in hydrodynamic size or thermal stability across this range, indicating that Ab1-scFv1 maintains its structural integrity and does not exhibit concentration-dependent aggregation under these conditions.

These findings suggest that Ab1-scFv1 is biophysically stable across a broad concentration range, supporting its use in both low- and moderately high-concentration applications.

#### 4.2.4 Protein quantification and UV absorbance analysis

Protein concentrations were determined by measuring absorbance at 280 nm (A280) using a micro spectrophotometer, often referred to as nanodrop. At 280 nm, absorbance primarily arises from the aromatic amino acids tryptophan (W) and Tyrosine (Y), as well as disulfide-linked cysteine (C) residues (Edelhoch, 1967). Extinction coefficients for each constructs were calculated based on their amino acid sequences. The extinction coefficient at 280 nm (ε280) can be determined experimentally or calculated theoretically based on the amino acid sequence by using bioinformatic tools, such as the ExPASy ProtParam tool (Pace et al., 1995). Once the extinction coefficient is known, the protein concentration (C) in solution can be calculated by using Beer-Lambert law: A280 = ε280 x C x ι where ι corresponds to the path length of the cuvette in centimeters (Grimsley and Pace, 2003).

In addition to concentration measurement, the UV absorbance spectra were inspected near 230 nm to evaluate potential protein aggregation. Elevated absorbance in this region can indicate the presence of aggregated material (Bashir et al., 2024). None of the construct showed unusual spectral features at 230 nm, suggesting that significant aggregation was not present in the purified samples (Supplementary Figure S3).

#### 4.2.5 Impact of sample handling on protein quality

To evaluate the effect of standard protein handling steps on sample quality, we used DLS to assess the impact of centrifugation and freeze-thaw cycles on aggregation behaviour in selected constructs.

**FIGURE 3:**
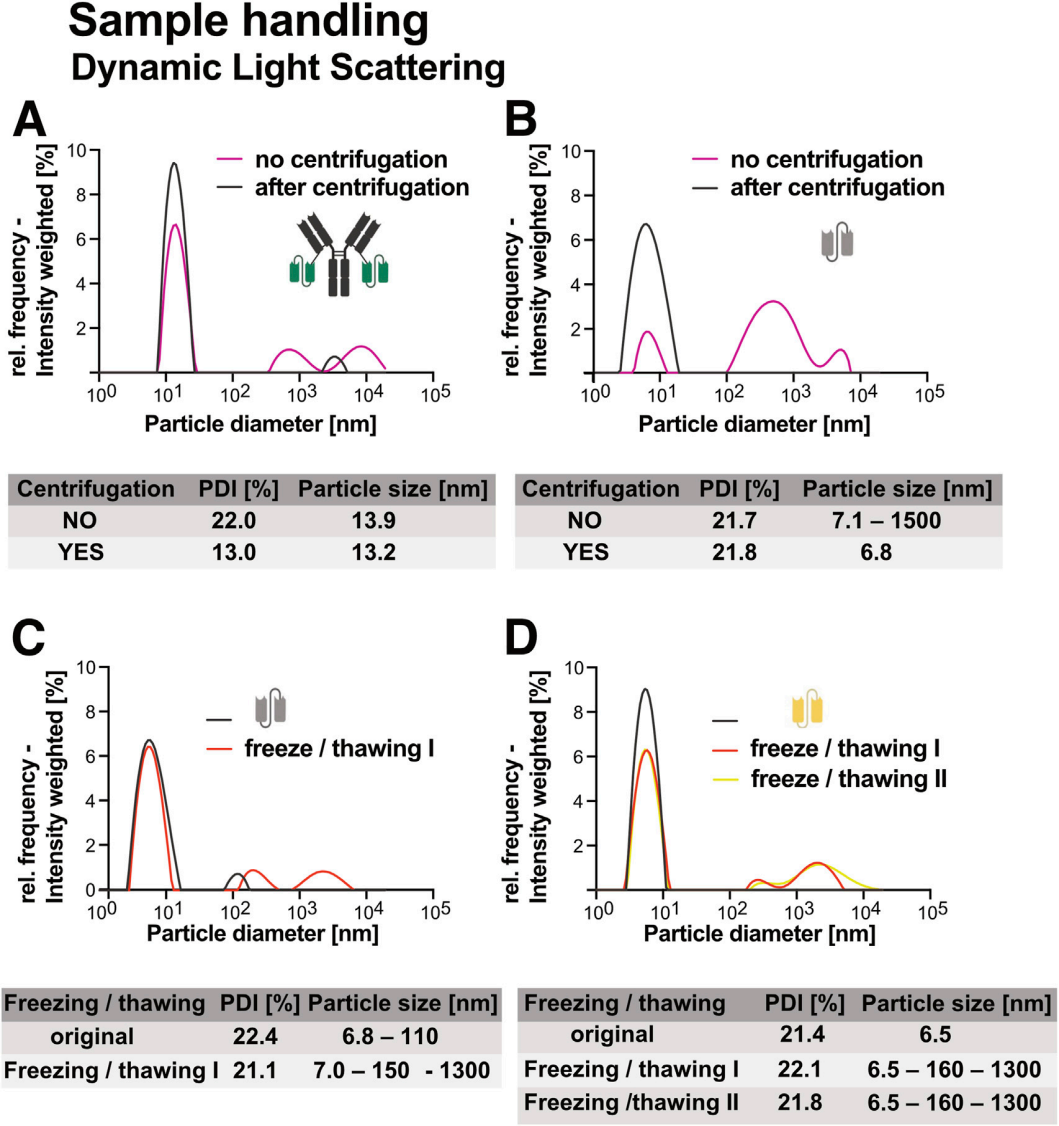
Effects of centrifugation and freeze-thaw cycles on dynamic light scattering (DLS) measurements of recombinant proteins. Particle size and polydispersity index (PDI) measurements before and after centrifugation illustrate the impact of centrifugation on sample homogeneity for **(A)** Ab1-scFv1 and **(B)** scFv3. The effects of freeze-thaw treatment on protein stability and aggregation are shown by changes in particle size and PDI for **(C)** scFv3 and **(D)** scFv4. All proteins were analyzed at a concentration of 1 mg/mL.

Centrifugation prior to use, significantly improved sample homogeneity. For both Ab1-scFv1 (Figure 3A) and scFv3 (Figure 3B), a 5 minute centrifugation at 10,000 x g (4 °C) reduced the presence of high-molecular weight species. This was reflected in a decrease in polydispersity index (PDI), indicating a shift toward more monodisperse populations. Based on these results, a brief centrifugation step is recommended as part of routine protein preparations. To ensure accurate dosing, A280 measurements should be repeated after centrifugation, as the process can result in concentration changes.

Freeze-thaw stability was evaluated for scFv3 (Figure 3C) and scFv4 (Figure 3D). Both constructs showed increased aggregation following one or more freeze-thaw cycles, as indicated by the emergence of larger particle populations. These results underscore the importance of minimizing freeze-thaw events, particularly for aggregation-prone proteins (Pang et al., 2023). Together, these findings highlight the value of simple preparatory steps, such as centrifugation and aliquoting, in maintaining sample quality and reducing variability in downstream experiments.

To further reduce aggregation and degradation risks, proteins should be stored in appropriately sized aliquots to avoid repeated freezing and thawing. Long-term storage at −80 °C is preferred, as proteins kept at −20 °C are more susceptible to chemical modifications, including oxidation (e.g., methionine and cystein), deamidation (asparagine and glutamine) and proteolytic degradation if residual protease activity is present (Černý et al., 2013; Powell et al., 2002; Richards, 1997). These modifications occur more slowly or are largely suppressed at −80 °C, making it the storage condition of choice for sensitive constructs.

### 4.3 Analysis of protein purity

Assessing protein purity is a critical step following purification and is tailored to the intended downstream applications. While minor impurities may be acceptable in some *in vitro* assays, higher purity is essential for studies involving cells or animals. Particular attention must be given to aggregates and contaminants such as endotoxins, as these can significantly impact biological outcomes (Malyala and Singh, 2008; Gorman and Golovanov, 2022).

#### 4.3.1 Endotoxin levels of purified proteins

Endotoxins, primarily lipopolysaccharides (LPS) from the outer membrane of Gram-negative bacterial sources, can trigger strong immune responses even at low concentrations. To prevent interference with cell-based or *in vivo* experiments, all expression and purification steps should be performed under endotoxin-minimizing conditions by using single-use, endotoxin-free plastic consumables throughout the entire workflow.

Endotoxin levels can be assessed using the Limulus amoebocyte lysate (LAL) test (Gorman and Golovanov, 2022). This assay is based on the clotting reaction of amoebocyte lysate derived from the horseshoe crab (Limunus polyphemus), which is highly sensitive to the presence of endotoxins (Iwanaga, 2007). Endotoxin units are reported per milligram protein (EU/mg). A commonly accepted threshold for *in vivo* work in mice is <0.1 EU/µg protein (100 EU/mg protein), stricter limits may apply depending on application. Inclusion of LAL data ensures experimental reproducibility and minimizes risk of immune activation (Malyala and Singh, 2008; Jeong et al., 2025). Specific thresholds may vary depending on the route of administration and regulatory guidelines (American Pharmaceutical Review, 2018).

#### 4.3.2 Determination of purity of recombinant proteins by SDS-PAGE

**FIGURE 4:**
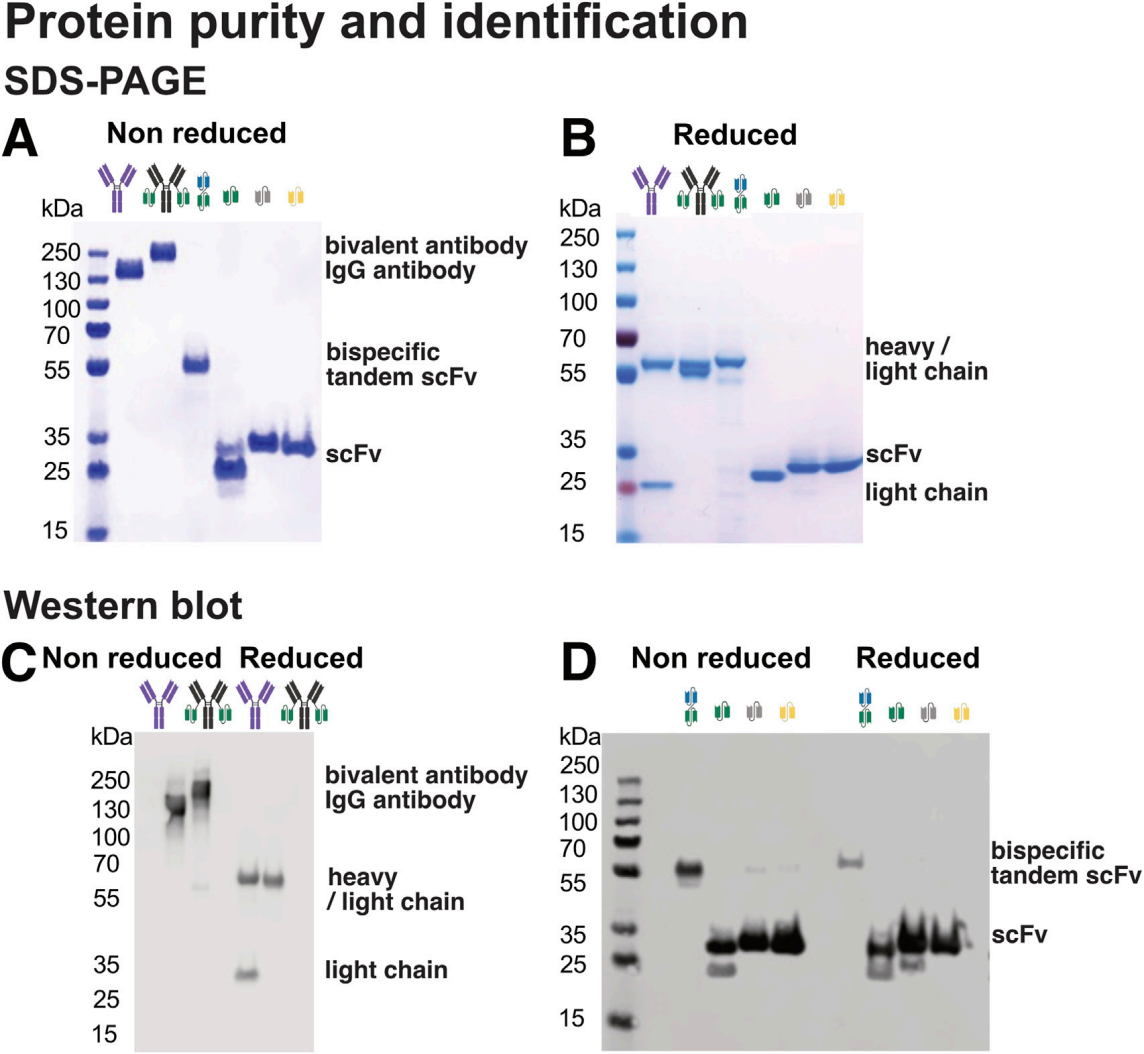
Design and purity of antibodies, multivalent antibodies, and antibody fragments used in this study. **(A)** SDS-PAGE analysis under non-reducing conditions, preserving disulfide bonds and allowing detection of intact antibody complexes. **(B)** SDS-PAGE under reducing conditions using DTT to break disulfide bonds, enabling separation and visualization of individual antibody chains. **(C)** Western blot analysis using an anti-IgG antibody to detect IgG-based antibody constructs and fragments. **(D)** Western blot analysis using an anti-His-tag antibody to detect His-tagged recombinant proteins.

To evaluate sample purity and subunit composition, all recombinant proteins were analysed by SDS-PAGE under both non-reducing (Figure 4A) and reducing conditions (Figure 4B).

Under non-reducing conditions, intact antibodies such as Ab1 migrated as expected, with limited evidence of degradation products or multimers. The fusion construct Ab1-scFv1 showed a similar migration pattern. Under reducing conditions, Ab1 separated into heavy and light chains, while Ab1-scFv1 displayed co-migrating heavy and light chains due to the increased molecular weight of the fused scFv domain. This resulted in both chains appearing at nearly identical positions in the gel, consistent with their design.

This shift in migration highlights the importance of reducing disulfide bonds, particularly in antibodies, where covalent disulfide bridges stabilize quaternary structure and link heavy and light chains (McAuley et al., 2008; Fass and Thorpe, 2018; Trivedi et al., 2009). Reducing agents such as DTT, β-mercaptoethanol, or tris(2-carboxyethyl) phosphine (TCEP) are used to disrupt these bonds and resolve individual subunits during SDS-PAGE (Han and Han, 1994).

Single-chain fragments (scFv1, scFv3, and scFv4) also showed expected migration patterns under both reducing and non-reducing conditions (Figures 4A,B). Notably, for scFv1, a minor high-molecular weight species observed under non-reducing conditions was no longer present under reducing conditions, suggesting the presence of disulfide-linked multimers.

While SDS-PAGE provided a general overview of purity and molecular weight, it may underestimate the presence of very large aggregates or very small contaminants due to gel resolution limits (Gallagher, 1999). Therefore, complementary analyses (such as mass photometry, DLS and SEC) were used for a more complete characterization.

### 4.4 Protein identification and confirmation

To confirm the identity of the expressed proteins and validate SDS-PAGE results, both mass spectrometry (MS) or antibody-specific detection with Western blot can be employed (Pillai-Kastoori et al., 2020; Mahmood and Yang, 2012).

MS enables unambiguous identification of the target proteins using only small amount of sample and is well suited for identifying small proteins and peptides making it a powerful tool for comprehensive analysis (Szabo and Janaky, 2015).

Figures 4C,D show Western blotting results with both anti-IgG and anti-His antibodies, which confirmed the presence of the target recombinant proteins under both non-reducing and reducing conditions. Notably, additional higher molecular weight bands were observed for scFv3 and scFv4 under non reducing conditions (Figure 4D), suggesting the formation of multimeric species not detectable by SDS-PAGE alone (Figure 4A). These findings highlight the importance of orthogonal detection methods for accurate protein characterization, particularly in cases where protein multimerization may affect function or interpretation of experimental results.

#### 4.4.1 Quantification of protein purity using densitometry

**FIGURE 5:**
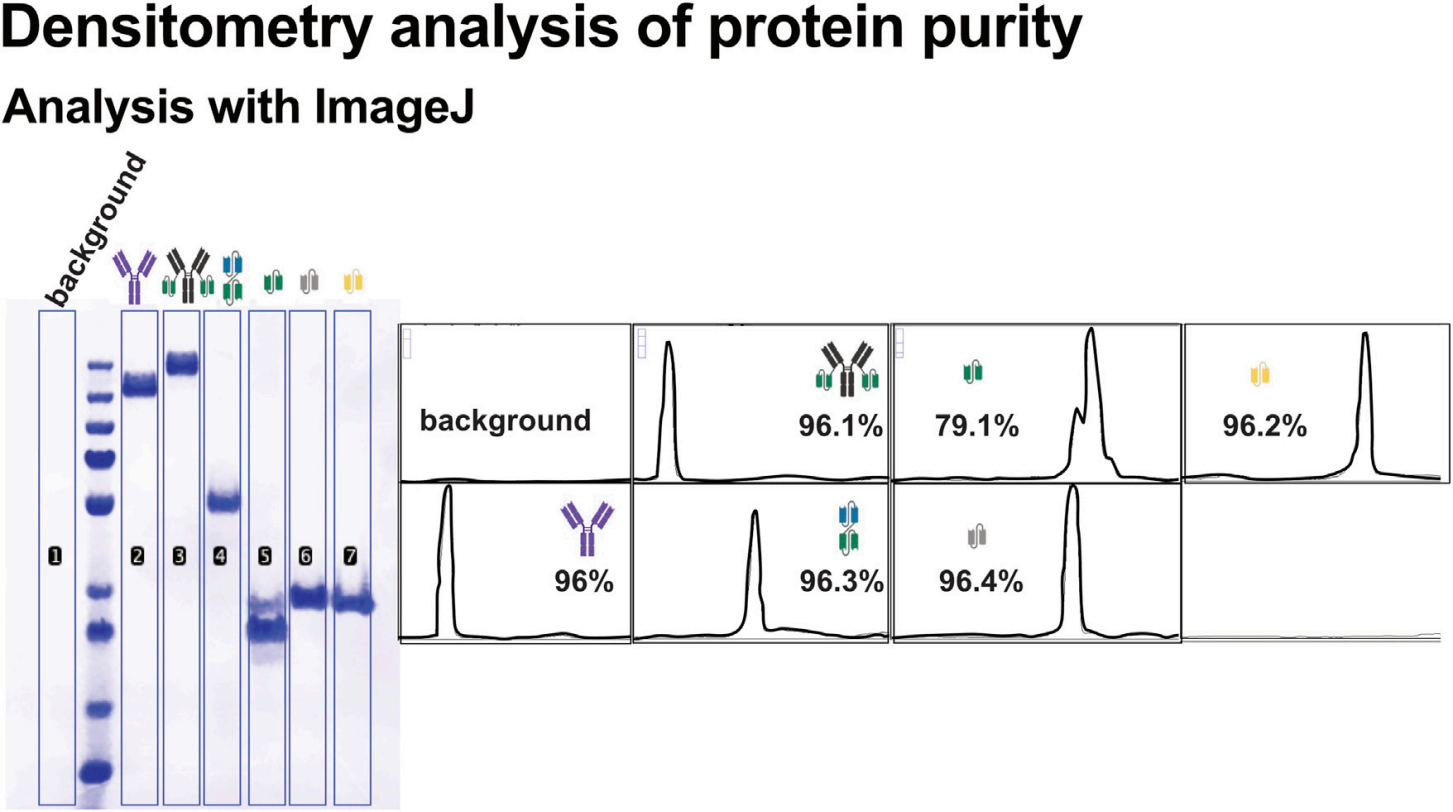
Densitometric analysis of recombinant protein purity using ImageJ. Band intensities from SDS-PAGE gels were quantified using ImageJ to evaluate the purity of the recombinant proteins. Background subtraction was applied to all measurements. Purity was calculated as a percentage (%) of the total lane intensity, and the calculated purity value is shown for each protein. Corresponding schematic representations of the protein constructs are included alongside the densitometry results.

To assess purity of recombinant protein preparations, densitometric analysis of Coomassie-stained SDS-PAGE gels (Figure 4A) was performed using ImageJ. Band intensities were quantified, and the proportion of the target protein relative to the total lane intensity was calculated to estimate sample purity (Figure 5).

Most recombinant proteins displayed high purity, with the dominant band corresponding to the expected molecular weight. In contrast, scFv1 exhibited a more heterogeneous band pattern, including prominent lower molecular weight species. This suggests the expression of truncated variants. Such heterogeneity may impact downstream applications and highlights the importance of purity assessment beyond visual inspection.

While SDS-PAGE provides a comprehensive view of total protein content, similar densitometric analysis can also be performed on Western blot images. This can be useful for comparing expression levels, detection of degradation products or multimerization of the recombinant proteins (Alonso Villela et al., 2020). However, this is limited to proteins being recognized by the antibody and may not reveal non-target impurities, making SDS-PAGE the preferred method for estimating overall sample purity.

This semi-quantitative approach enables reliable comparison of protein quality across different constructs and purification batches, offering insight into the consistency and integrity of recombinant protein preparations.

### 4.5 Analysis of protein thermal stability

Thermal unfolding profiles obtained by nano differential scanning fluorimetry (nanoDSF) not only reflect overall protein stability but also provide insights into unfolding cooperativity and inter-domain interactions within fusion proteins. In multi-domain proteins, several distinct unfolding patterns can occur: (1) independent domain unfolding, where separate transitions suggest minimal interaction between domains; (2) cooperative unfolding with a single intermediate transition, indicating potential stabilization or destabilization effects between domains; (3) global destabilization, where a single low-temperature transition implies steric interference or misfolding due to domain fusion; and (4) irregular, broad transitions that may indicate partial misfolding or aggregation, especially when accompanied by increased light scattering. These unfolding behaviours can be influenced by factors such as linker composition, domain orientation, tryptophan distribution, and domain folding dependencies (Wen et al., 2020; Hamuro et al., 2021; Kim et al., 2021).

**FIGURE 6:**
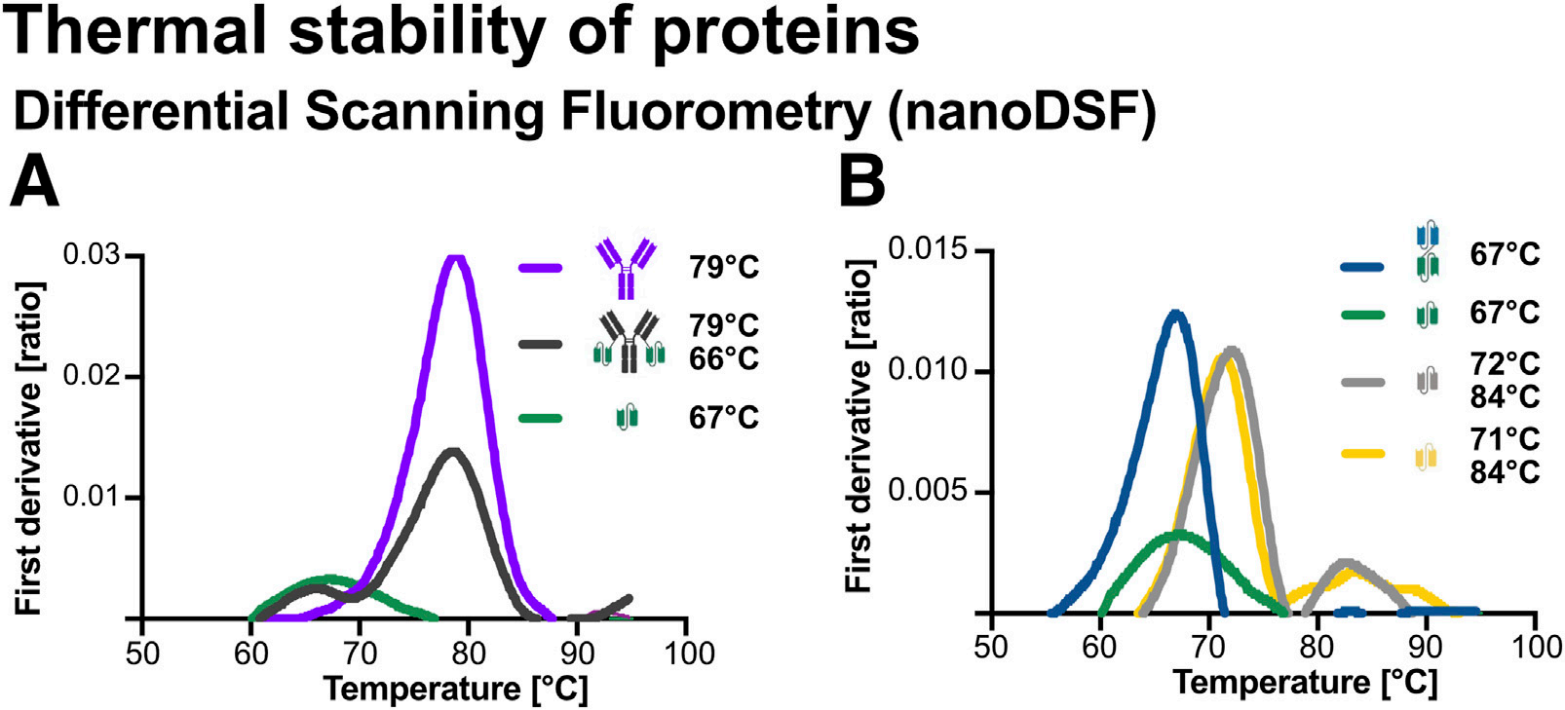
Thermal stability analysis of recombinant proteins using nano differential scanning fluorimetry (nanoDSF). Thermal unfolding profiles were obtained using a Prometheus Panta instrument (NanoTemper Technologies). Protein samples (2 μM) were loaded into glass capillaries and subjected to a linear temperature gradient from 30 °C to 95 °C. **(A)** First derivative of the thermal unfolding curves and corresponding inflection temperatures (Ti) for Ab1 (79 °C), Ab1‐scFv1 (66 °C and 79 °C), and scFv1 (67 °C). **(B)** First derivative curves and Ti values for bi‐scFv2‐scFv1 (67 °C), scFv1 (67 °C), scFv3 (72 °C and 84 °C), and scFv4 (71 °C and 84 °C).

To assess the thermal stability and domain behaviour of the recombinant proteins, nanoDSF was performed using the Prometheus Panta platform (NanoTemper Technologies). This method monitors changes in the intrinsic fluorescence of aromatic amino acids (tryptophan and tyrosine) during a controlled temperature ramp, allowing detection of unfolding transitions through inflection temperature (Ti) in the fluorescence ratio derivative (Temel et al., 2016; Llowarch et al., 2023). Thermal unfolding profiles were recorded between 30 °C–95 °C. As shown in Figure 6A, the fusion construct Ab1-scFv1 displays two distinct unfolding transitions at 66 °C and 79 °C. These match the Ti values of its individual components, scFv1 (66 °C) and Ab1 (79 °C), indicating that both domains unfold independently within the fusion construct.

Similarly, scFv3 and scFv4 (Figure 6B), showed two Ti’s at approximetely 72 °C and 84 °C (Figure 6B), indicating the presence of two thermally distinct structural regions or domain-like behaviour. In contrast, scFv1 and bi-scFv2-scFv1 (Figure 6B) displayed single sharp transitions, consistent with cooperative unfolding of a single domain.

These results demonstrate varying thermal stability profiles across the constructs, with multi-domain proteins showing discrete, indepentently unfolding regions. Importantly, all constructs remained stable well above physiological temperature (37 °C), supporting their suitability for therapeutic or diagnostic applications.

### 4.6 Multimerization and aggregation status of proteins

Protein multimerization and aggregation are distinct but often overlapping phenomena that can significantly affect experimental outcomes and protein functionality. Multimerization refers to the non-covalent assembly of functional protein complexes, whereas aggregation typically involves non-specific, often irreversible clustering of misfolded or partially folded proteins. Both can alter binding characteristics, particularly through avidity effects; and in therapeutic applications, they may trigger undesired immune reactions (Ratanji et al., 2014; Pang et al., 2023).

Determining whether a protein exists as a monomer, multimer or aggregate in solution is essential, as convential methods like SDS-PAGE may fail to capture multimeric statues due to dissociation under denaturing conditions (Gallagher, 1999). Therefore, orthogonal solution-based methods are required to accurately characterize the oligomeric state and aggregation behaviour of proteins.

In this study we employed three complementary biophysical techniques, mass photometry, dynamic light scattering (DLS), and size exclusion chromatography (SEC), to assess the multimerization and aggregation states of the recombinant proteins under native conditions. These analyses provide insights into sample homogeneity, particle size distribution, and potential multimer formation that may not be apparent from gel-based methods alone.

#### 4.6.1 Analysis of multimerization and aggregation status of protein by mass photometry

Mass photometry enables label-free analysis of proteins in solution by measuring light scattering from individual molecules as they interact with a glass surface (Wu and Piszczek, 2021a; Wu and Piszczek, 2021b; Young et al., 2018). Molecular masses are determined by comparing the scattering signal to calibrated standards (Schiller, 2021; Soltermann et al., 2020).

**FIGURE 7:**
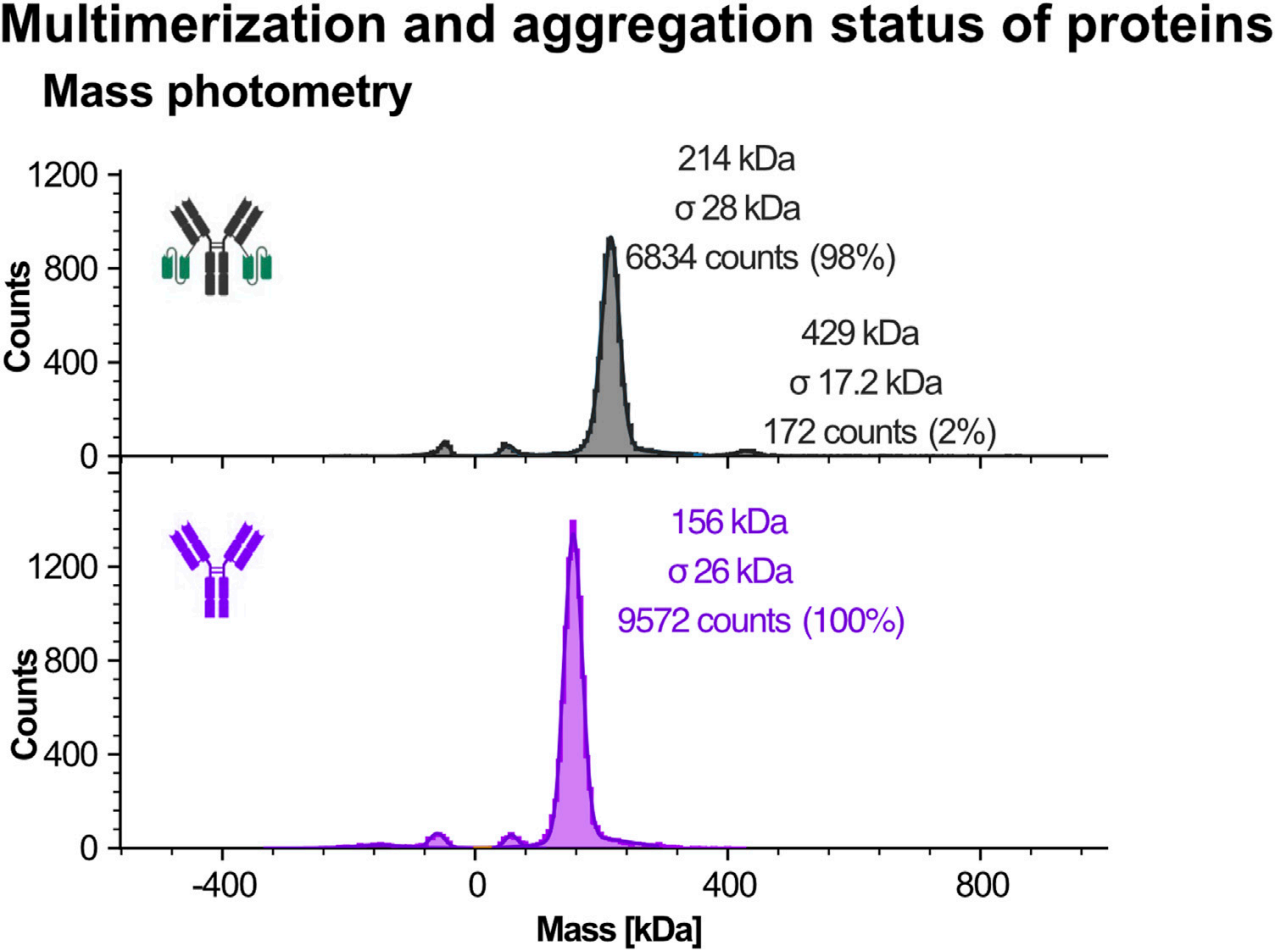
Mass photometry analysis of recombinant protein purity using the Refeyn instrument. Mass photometry was used to assess the purity and molecular mass of larger recombinant proteins, Ab1 and Ab1-scFv1. This technique measures light scattering signals generated when individual molecules interact with a glass surface, with signal intensity correlating directly to molecular mass. Due to detection limits (typically >30-40 kDa), only Ab1 and Ab1-scFv1 were analyzed. Schematic representations of the respective protein constructs are shown alongside the data. Protein purity was assessed using two commercial IgG calibrants: one as a monomer standard, and one containing both monomer and dimer species. Symmetrical peaks mirrored around 0 kDa represent typical artifacts of mass photometry (e.g., buffer or surface-related signals) and were excluded from analysis.

Due to the instrument’s sensitivity threshold, typically around 40–50 kDa, only the larger recombinant proteins Ab1 and Ab1-scFv1 were suitable for analysis. As shown in Figure 7, Ab1-scFv1 displayed a primary peak corresponding to the expected monomeric form, along with a minor population (approximately 2%) consistent with dimeric species. In contrast Ab1 appeared exclusively monomeric under the same conditions.

These findings suggest that fusion of scFv1 to Ab1 slightly increases the tendency for multimerization, possibly due to conformational changes introduced by the fusion. While the proportion of dimers is low, such multimeric species could still influence binding activity or downstream performance in sensitive applications.

#### 4.6.2 Analysis of multimerization and aggregation status using dynamic light scattering

**FIGURE 8:**
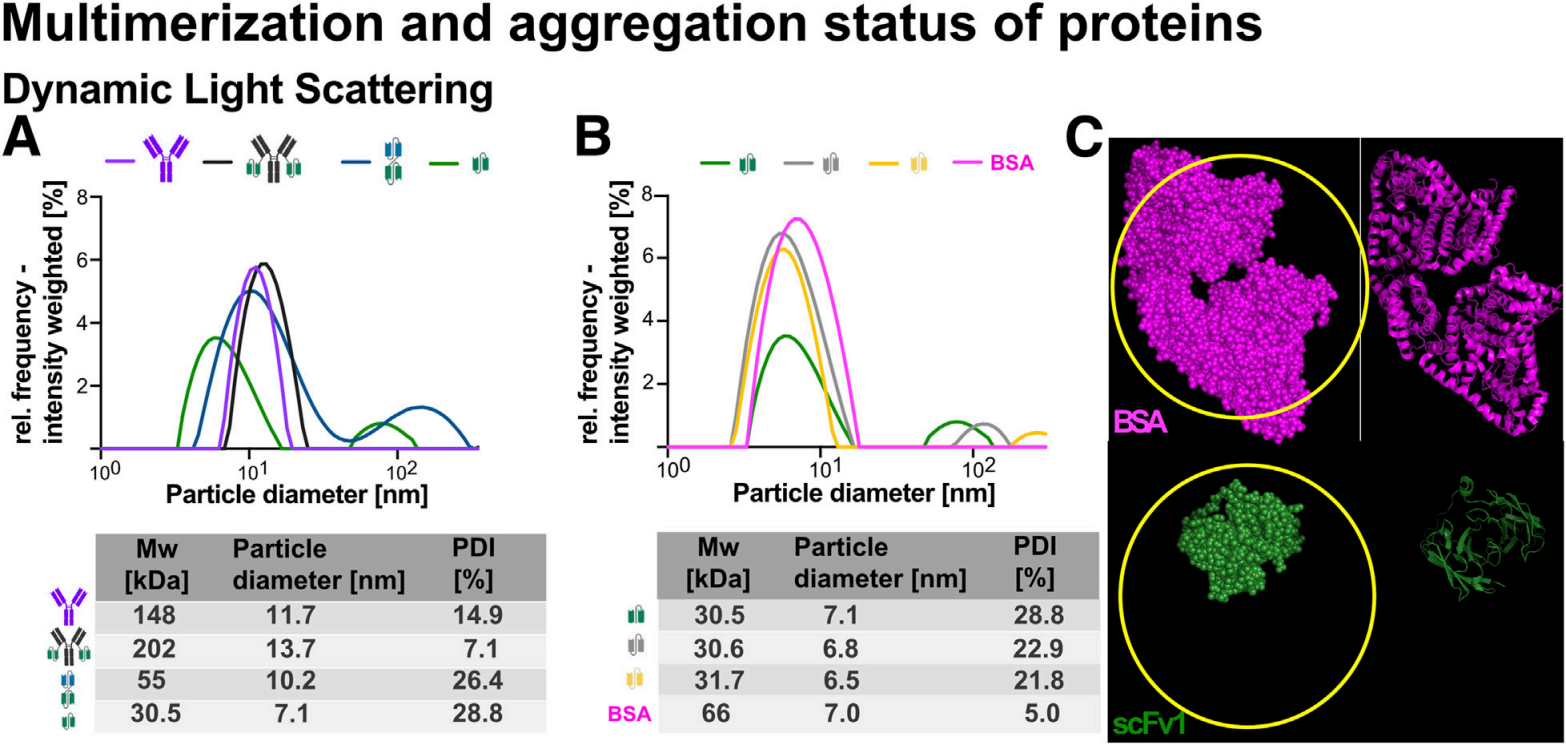
Dynamic light scattering (DLS) analysis of recombinant proteins. Recombinant proteins were analyzed at 2 μM concentration using DLS to assess particle size distribution and aggregation behavior. **(A)** DLS profiles for Ab1, Ab1‐scFv1, bi‐scFv2‐scFv1, and scFv1. **(B)** DLS profiles for scFv1, scFv3, scFv4, and bovine serum albumin (BSA) monomer as a reference. Schematic representations of each protein are included in the graphs. Particle diameter (nm) was determined using an intensity-weighted model, with values summarized in a table below the graphs. The polydispersity index (PDI) was calculated for each sample as a measure of sample heterogeneity. Ab1, Ab1‐scFv1, and BSA monomer exhibited low PDI values, indicating predominantly monodisperse or slightly multidisperse populations. In contrast, the antibody fragments (bi‐scFv2‐scFv1, scFv1, scFv3, and scFv4) showed higher PDI values and an additional particle size peak around 100 nm (not included in the table), suggesting aggregation or multimerization. **(C)** PyMOL visualization comparing the AlphaFold2‐predicted structure of scFv1 with the crystal structure of BSA monomer (PDB ID: 3V03). This highlights that DLS estimates hydrodynamic diameter based on diffusion of idealized spherical particles; thus, similar particle sizes may not directly reflect similar molecular weights. The apparent similarity in particle diameter between scFv1 and BSA monomer suggests potential dimerization of scFv1, as indicated within the highlighted “yellow ring” region.

To assess particle size distribution and sample heterogeneity, dynamic light scattering (DLS) was performed using intensity-weighted measurements, which emphasizes larger species and is best suited for identifying the presence of aggregates and multimeric species (Figures 8A,B). DLS measures the hydrodynamic diameter of particles or molecules by analyzing the fluctuations in scattered light caused by their Brownian motion in solution (Maguire et al., 2018). Other distribution types exist, such as volume-weighted (which offers a more balanced view for formulation studies) and number-weighted size (which highlights smaller particles). From this data, hydrodynamic diameter and polydispersity index (PDI) can be calculated to evaluate the monodispersity of each sample (Stetefeld et al., 2016).

Ab1 and Ab1-scFv1 both showed monodisperse profiles with low PDI values (14.9% and 7.1%, respectively), indicating predominantly uniform populations in solution (Figure 8A). The slight increase in hydrodynamic diameter from 11.7 nm (Ab1) to 13.7 nm (Ab1-scFv1) reflects the expected size change upon fusion with an scFv domain (Nguyen et al., 2025). The bi-scFv2-scFv1 construct exhibited a diameter of approximately 10.2 nm (Figure 8A), similar to the antibody formats. This suggests an extended, non-globular conformation. However, its high PDI (above 20%) indicates a heterogenous mixture, possibly due to conformational variability or multimerization.

The scFv variants (scFv1, scFv3, scFv4) displayed smaller hydrodynamic diameters (approximately 7 nm). For comparison, monomeric bovine serum albumin (BSA), a well-characterized globular reference protein with known diameter of approximately 7 nm was included (Stetefeld et al., 2016) (Figure 8B). Despite their smaller molecular weight (32 kDa), the scFv exhibited similar diameters to BSA, suggesting that DLS, which assumes spherical particles, may overestimate the size of elongated or asymmetric molecules. Structural models further support this, showing that multiple scFv molecules could theoretically fit within the volume occupied by a BSA-sized particle (Figure 8C).

Importantly, all scFv variants exhibited relatively high polydispersity index (PDI) values (21.8%–28.8%), indicating a heterogeneous population likely resulting from multimerization or aggregation. This is further supported by the appearance of a secondary peak around 100 nm in their size distributions, indicative of larger species not present in Ab1, Ab1-scFv1 or BSA.

In summary, Ab1, Ab1-scFv1, and BSA were predominantly monodisperse, while the scFv variants and bi-scFv2-scFv1 showed higher polydispersity and signs of multimerization and aggregation.

#### 4.6.3 Analysis of oligomeric state using size exclusion chromatography

SEC was used as a third, orthogonal method to assess the oligomeric state and aggregation behaviour of the recombinant proteins. SEC separates proteins based on their hydrodynamic volume, allowing for the identification of monomers, dimers, and higher-order oligomers under native, non-denaturing conditions (Fekete et al., 2014; Folta-Stogniew et al., 2006). Unlike SDS-PAGE, which can disrupt non-covalent interactions, SEC provides a more accurate representation of native multimeric states in solution, even though detection for IgG formats is often limited to dimers and oligomers as traces of large aggregates are often not detected (Ahrer et al., 2003).

**FIGURE 9:**
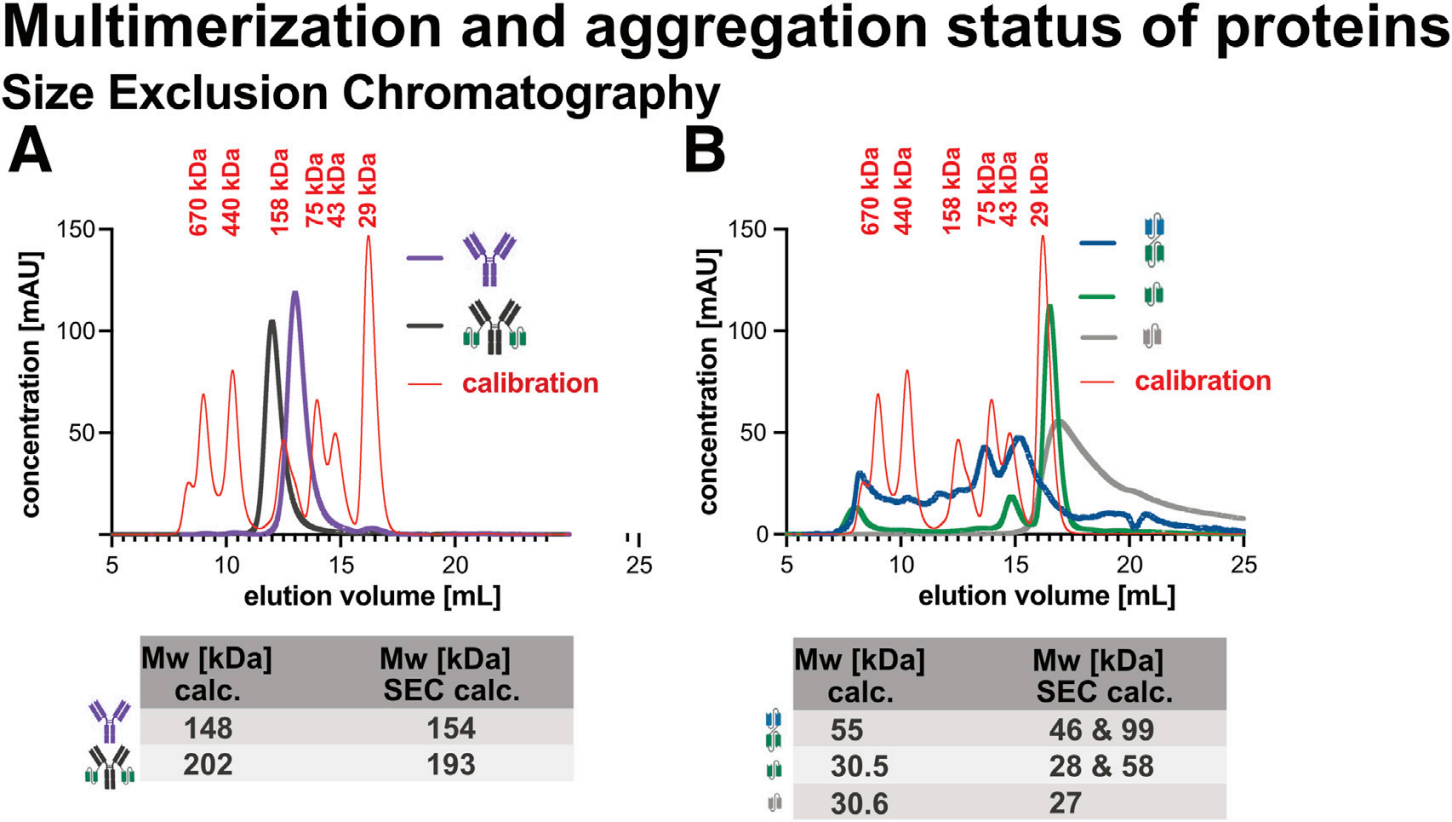
Size exclusion chromatography (SEC) analysis of recombinant proteins. Recombinant proteins were analyzed by SEC to assess molecular weight and sample homogeneity. **(A)** SEC profiles of Ab1 and Ab1‐scFv1. **(B)** SEC profiles of bi-scFv2-scFv1, scFv1, and scFv3. Each sample (50 μg) was run on a Superdex 200 Increase 10/300 GL column (Cytiva), and retention times were compared to a molecular weight calibrant. Both the theoretical and SEC-determined molecular weights are summarized in a table below the chromatograms. Ab1 and Ab1‐scFv1 eluted as single, symmetric peaks consistent with their expected monomeric forms and showed no evidence of aggregation or multimerization. In contrast, the antibody fragments, bi‐scFv2‐scFv1, scFv1, and scFv3, displayed signs of aggregation and higher-order multimer formation. The SEC profile of scFv3, in particular, revealed a broad and asymmetric peak, indicating the presence of heterogeneous species with a range of molecular weights.

As shown in Figure 9A, both Ab1 and Ab1-scFv1 eluted as single, symmetrical peaks corresponding to their expected molecular weights, with no detectable high-molecular weight species. This indicates that these full-length antibody constructs are predominantly monomeric and structurally homogenous in solution.

In contrast, bi-scFv2-scFv1, scFv1, and scFv3 (Figure 9B) showed additional earlier-eluting peaks, consistent with the presence of higher-order species or aggregates. Furthermore, deviations between the expected and apparent molecular weights suggest non-globular or self-associated conformations. These findings align with the DLS data and support the conclusion that smaller fragments are more prone to multimerization and aggregation, likely due to their exposed interaction surfaces or conformational flexibility.

### 4.7 Additional methods for protein characterization

#### 4.7.1 Is the protein refoldable?

The ability of a protein to refold after thermal denaturation provides insights into its folding autonomy and structural resilience. This characteristic is often seen in single-domain proteins, while multi-domain proteins typically require cellular chaperones to fold correctly. *In vitro*, the absence of such support can lead to misfolding or aggregation, especially upon thermal stress.

A simple way to assess refolding capacity, is thermal cycling using nanoDSF. Proteins are heated to 95 °C to induce unfolding, cooled to room temperature and then re-analysed. If the post-heating unfolding profile resembles the original (pre-heating) profile, it suggests successful refolding. Conversely, a flat or altered profile indicates loss of native structure (not shown).

While structural characterisation is essential for understanding protein function, stability, and interactions, high-resolution techniques such as X-ray crystallography and nuclear magnetic resonance (NMR) spectroscopy were not used due to their technical and material demands. Instead, we employed circular dichroism (CD) spectroscopy as a rapid and accessible method to assess secondary structure and thermal stability (Greenfield, 2015; Kelly et al., 2005; Chemes et al., 2012).

##### 4.7.1.1 Analysis of structure using circular dichroism

CD spectroscopy provides information on a-helices, ß-sheets, or disordered content by measuring the differential absorbance of right- and left-circularly polarized light by chiral molecules (Kelly et al., 2005; Rodger and Marshall, 2021). CD spectroscopy is particularly informative for fusion proteins, as it reveals both secondary structure content and thermal unfolding behaviour, which can uncover inter-domain interactions. Key interpretive features include: (1) multi-phase unfolding curves, suggesting independently folding domains; (2) single cooperative transitions, indicating coupled domain behaviour; (3) shifted melting temperatures compared to individual domains, reflecting stabilizing or destabilizing inter-domain interactions; (4) altered secondary structure spectra, suggesting conformational changes due to fusion; and (5) irreversible folding or poor signal recovery, indicating aggregation or misfolding (Martin and Schilstra, 2008; Atsavapranee et al., 2021; Budyak et al., 2024; Trolese et al., 2025).

**FIGURE 10:**
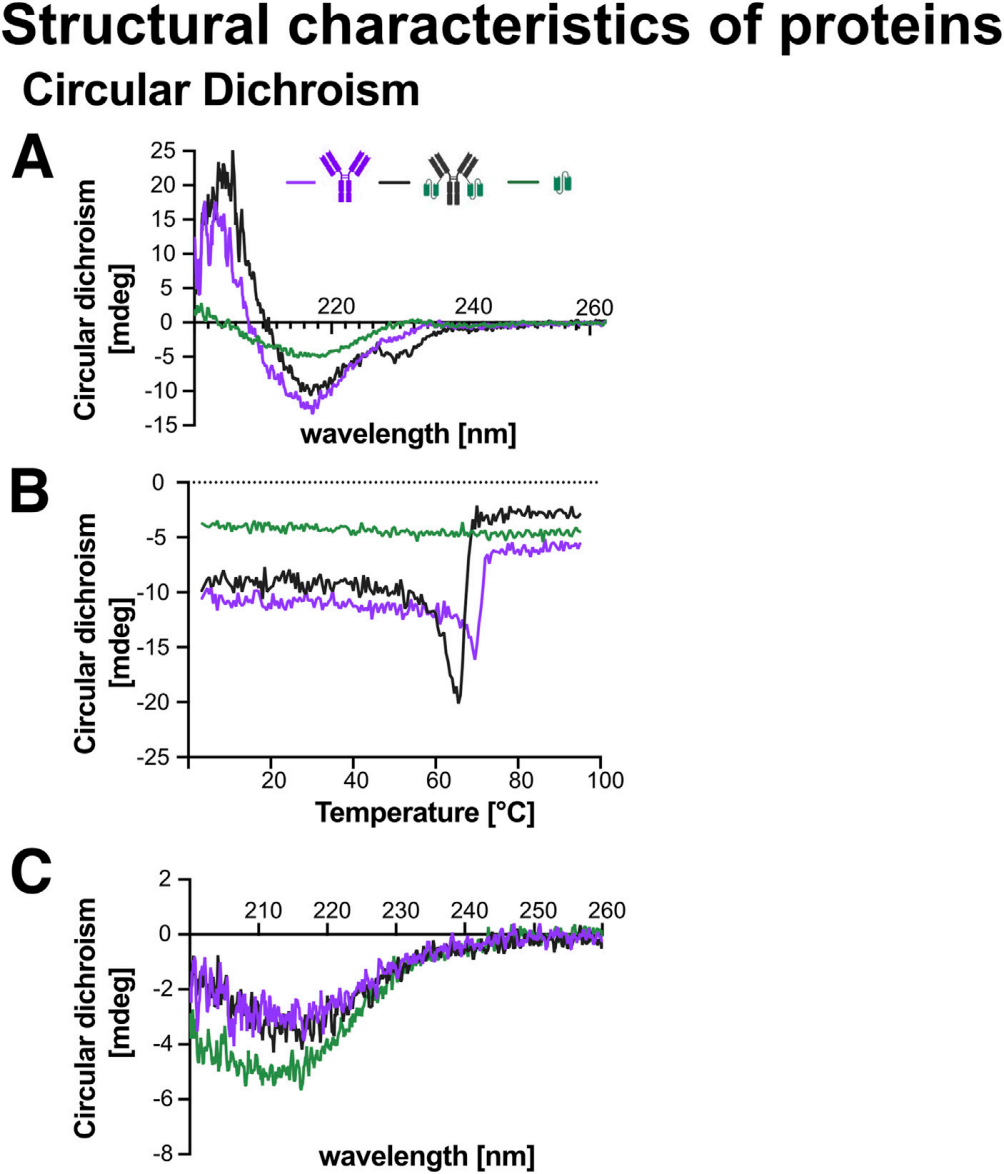
Circular dichroism (CD) analysis of the secondary structure and thermal stability of recombinant proteins. CD spectroscopy was performed on Ab1, Ab1-scFv1, and scFv1 at a concentration of 2 μM to assess secondary structure and thermal folding behavior. Schematic representations of each protein are included in the graphs. **(A)** CD spectra recorded at 25 °C from 200–260 nm revealed the characteristic secondary structure of each protein. The wavelengths corresponding to structural minima were identified as 217 nm for Ab1, 215 nm for Ab1-scFv1, and 218 nm for scFv1. **(B)** Melting temperature (Tmelt) analysis was conducted by monitoring the change in ellipticity at the respective wavelengths (217, 215, and 218 nm) during a linear temperature increase, allowing assessment of protein thermal stability. **(C)** A second CD spectrum was recorded at 25 °C after the Tmelt analysis to evaluate potential changes or loss in secondary structure due to thermal unfolding and refolding.

CD-spectra were recorded between 200–260 nm for Ab1, Ab1-scFv1, and scFv1 to identify characteristic wavelengths suitable for thermal unfolding (Tmelt) analysis: 217 nm (Ab1), 215 nm (Ab1-scFv1), and 218 nm (scFv1) (Figure 10A). The spectral profiles of Ab1 and Ab1-scFv1 were consistent with predominantly ß-sheet structures, as expected for antibodies. This aligns with known secondary structure characteristics of immunoglobulin domains (Charles et al., 2001). Notably, Ab1-scFv1 exhibited an additional structural feature around 230 nm, potentially indicating a conformational change or additional secondary structure introduced by the scFv fusion.

Tmelt profiles revealed that Ab1 and Ab1-scFv1 maintained comparable thermal stability, whereas scFv1 alone showed a relatively flat profile, suggesting either a lack of defined secondary structure or a pre-existing unfolded or aggregated state under the tested conditions (Figure 10B).

To evaluate structural recovery, CD spectra were recorded post-Tmelt. scFv1 partially regained to its original spectrum, indicating partial refolding and partial restoration of structural integrity. In contrast, both Ab1 and Ab1-scFv1 displayed markedly altered post-heating spectra (Figure 10C), indicating an inability to refold under the tested conditions. These results are consistent with the expectations that multi-domain proteins are generally less capable of autonomous refolding in the absence of cellular machinery.

##### 4.7.1.2 Small-angle X-ray scattering (SAXS) to analyse structural properties

To investigate the overall shape and conformational properties of antibodies and their engineered constructs in solution, we employed small-angle X-ray scattering (SAXS), a powerful technique for low-resolution structural analysis of macromolecules in solution (König et al., 2017; Jacques and Trewhella, 2010; Tian et al., 2014).

**FIGURE 11:**
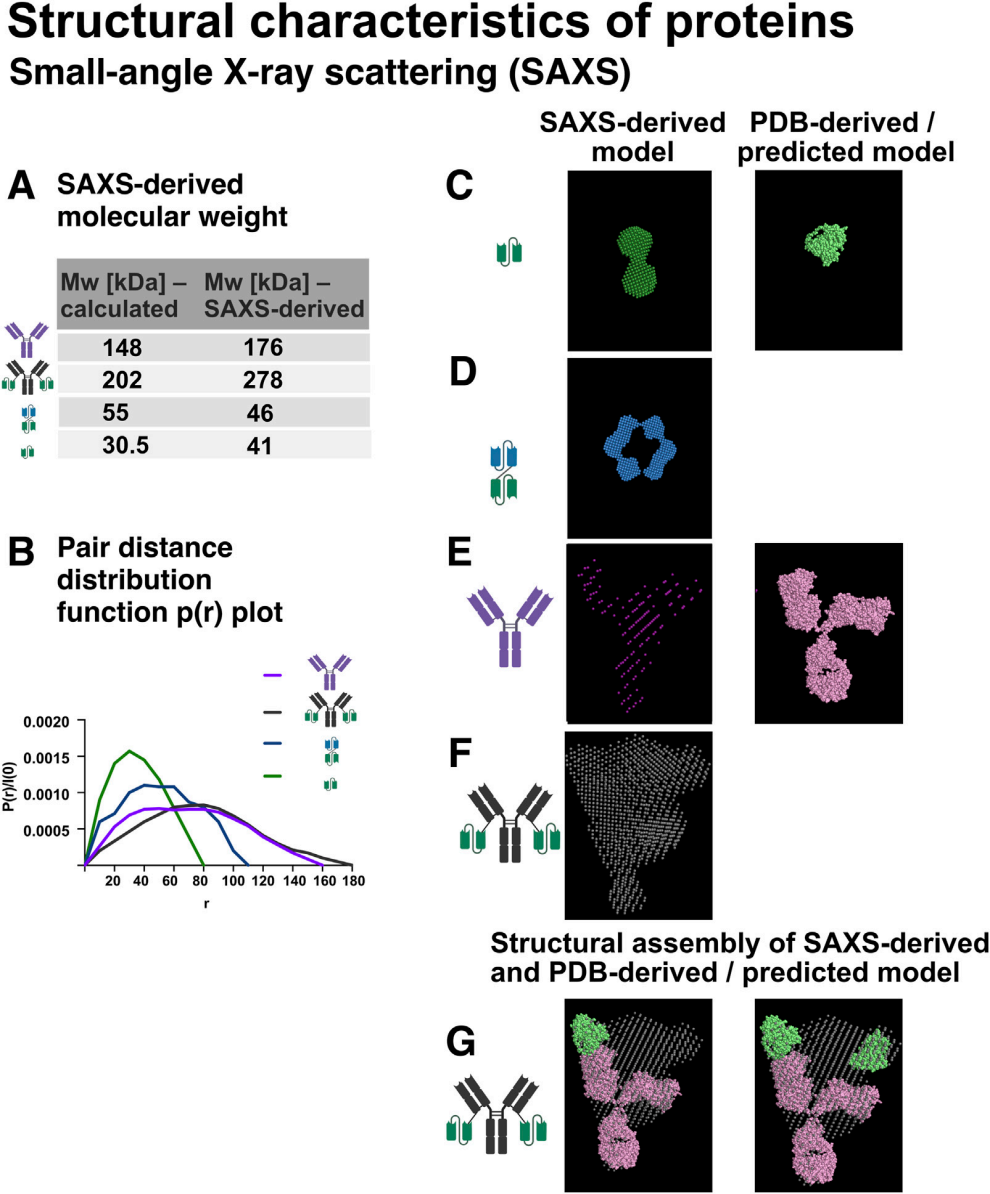
Small-angle X-ray scattering (SAXS) analysis of recombinant protein structure in solution. SAXS was employed to investigate the overall shape, size, and conformation of the recombinant proteins in solution. Schematic representations of the protein constructs are shown in the figure. **(A)** Molecular weight estimation based on SAXS data, performed using the software RAW. **(B)** Pair distance distribution function P(r) plots for Ab1, Ab1-scFv1, bi-scFv2-scFv1, and scFv1, derived via indirect Fourier transformation of the scattering curves. These plots provide insights into the overall shape and maximum particle dimension (D_max_). **(C)** SAXS-derived model of scFv1 (forest green), shown alongside the AlphaFold2-predicted structure (light green). **(D)** SAXS-generated model of bi-scFv2-scFv1 (blue). **(E)** SAXS model of Ab1 (magenta) overlaid with the crystal structure of IgG2a (PDB ID: 1igt, pink). **(F)** SAXS model of Ab1-scFv1 (grey). **(G)** Structural overlay comparing IgG2a (PDB ID: 1igt, pink), SAXS-derived Ab1-scFv1 model (grey), and the AlphaFold2-predicted structure of scFv1 (light green), highlighting differences in size, domain orientation, and overall conformation.

Molecular weight estimation based on extrapolated forward scattering intensity [I(0)], calculated using the program RAW, confirmed that Ab1, Ab1-scFv1, bi-scFv2-scFv1, and scFv1 were consistent with their theoretical molecular weights derived from their primary sequences, supporting sample integrity and validating the SAXS measurements (Figure 11A).

To further analyse the size and shape of the proteins in solution, pair distance distribution functions (P(r)) were generated for Ab1, Ab1-scFv1, bi-scFv2-scFv1, and scFv1 using the Fourier transformation of the SAXS data (Figure 11B). The P(r) function describes the distribution of intra-particle distances and provides insights into the molecule’s overall shape and internal structural organisation. From the P(r) curves, both the maximum particle dimension (Dmax) and the radius of gyration (Rg) were derived. Rg reflects the average mass distribution from the centre of the particle, while Dmax represents the longest distance between two points within the molecule, offering a complementary, model-independent size estimate. SAXS quality metrics including Guinier range analysis, X2-values (statistical fit) and Kratky plots are summarised in Supplementary Figure S4.

Broader P(r) curves, as observed for Ab1 and Ab1-scFv1 (Figure 11B), indicate an extended or flexible conformation, typical for full-length antibodies. In contrast, the narrower curves for scFv1 and bi-scFv2-scFv1 are consistent with more compact structures, although the bispecific construct still shows an elongated profile due to its engineered design.

To investigate the structural features of the antibody constructs and evaluate their conformational similarities to known or predicted reference structures, SAXS-based models were generated and overlaid with reference crystal structures or AlphaFold 2-predicted models. In Figure 11C, the SAXS-derived model of scFv1 (forest green) is compared with the AlphaFold 2-predicted structure of scFv1 (light green), used here as a reference scFv. The SAXS envelope accommodates two scFv-like domains, suggesting that scFv1 exists as a dimer in solution. The SAXS-based model of the bi-scFv2-scFv1 (blue, Figure 11D) also supports a dimeric state, with an extended conformation consistent with its dual-domain architecture.

For the full-length antibody Ab1, the SAXS-derived model (magenta, Figure 11E) was aligned with the crystal structure of mouse IgG2a (PDB ID: 1igt, pink). The comparison confirms the expected Y-shaped structure, with some deviations likely attributable to hinge region flexibility and SAXS capturing conformational variability in solution. The model of Ab1-scFv1 (grey, Figure 11F) maintains the core antibody shape but shows an additional density extending from each Fab arm, consistent with the fused scFv1 domain. This suggests that the scFv is structurally integrated into the antibody yet retains a degree of spatially flexibility, typical for fusion proteins.

Finally, Figure 11G presents an integrated overlay of the SAXS-derived models of Ab1-scFv1 (grey) with IgG2a (pink), along with the AlphaFold 2-predicted structure of scFv1 (light green). This composite view highlights the relative dimensions, domain orientations, and the impact of scFv fusion on the overall structure and spatial organisation of the constructs.

### 4.8 Visualization of proteins

Electron microscopy (EM) enables the direct visualization of large protein molecules, providing insight into their overall shape and structural contours. While EM lacks atomic-level resolution, unless advanced methods such as cryo-electron microscopy (cryo-EM) are employed, it remains a valuable tool for obtaining low-resolution structural information about macromolecular complexes (Jay et al., 2018; Zhang et al., 2015).

**FIGURE 12:**
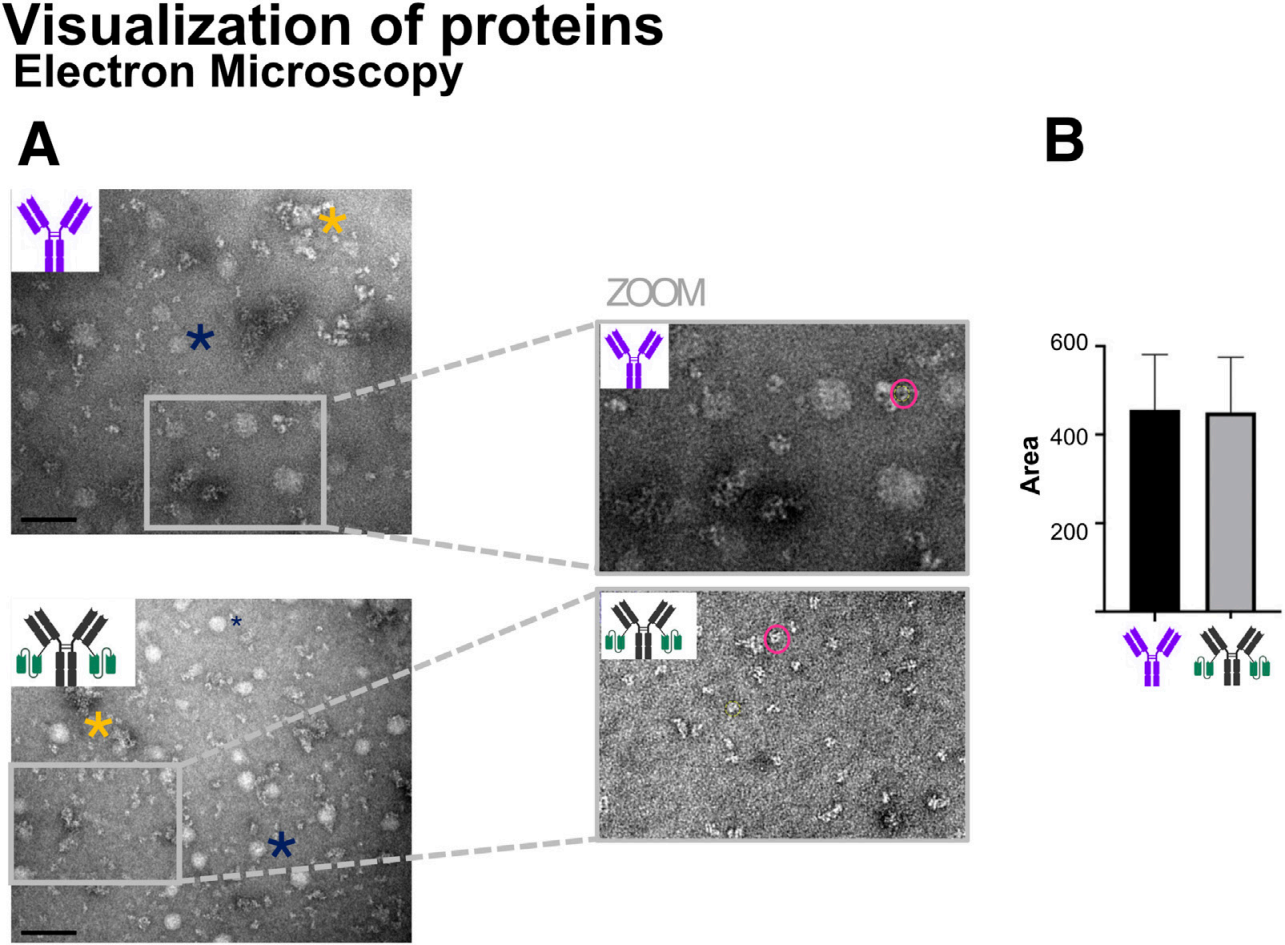
Transmission electron microscopy (TEM) visualization of antibody morphology. **(A)** TEM images of Ab1 and Ab1-scFv1 at 0.5 mg/mL (Ab1 = 3.3 μM; Ab1-scFv1 = 2.4 μM) reveal morphological features of the antibodies. The blue asterisks mark representative artifacts (circular white spots) attributed to buffer components in the preparation protocol, while yellow asterisks indicate antibody aggregates formed by clustering of multiple antibody molecules. Scale bar = 100 nm. Insets provide zoomed-in views for detailed observation of antibody structure. **(B)** Quantification of antibody particle area was performed on n = 40 particles per sample using ImageJ, with results summarized in the accompanying graph.

Figure 12A presents representative electron micrographs of Ab1 and Ab1-scFv1, while Figure 12B shows a semi-quantitative analysis of particle area measured based on ImageJ measurements. The data indicate no significant difference in particle size between Ab1 and Ab1-scFv1, suggesting that the addition of the scFv does not substantially alter the overall morphology and dimension of the antibody at the resolution accessible by EM.

Additionally, electron micrographs can be useful for identifying protein aggregation, which typically appears as irregular, dense, or clustered structures (Kumar et al., 2020). An example of such an aggregate is marked with a yellow asterisk in Figure 12A. However, aggregation was not observed as a general issue for the antibody samples analysed with this method.

### 4.9 Summary of analytical methods

To consolidate the analytical strategies used throughout this study, Table 1 presents a comparative summary of all experimental methods employed. This overview highlights the key advantages associated with each method, such as effectiveness and applicability in specific contexts.

**TABLE 1:** Summary of analytical methods highlighting sample requirements, resolution, data presentation, throughput, advantages and limitations.

Method	Purpose	Method description	Sample requirements	Resolution	Data presentation	Through-put	Advantages	Limitations
SDS PAGE	Protein purity Molecular weight	Gel electro-phoresis technique to separate proteins based on their molecular weight	Soluble protein samples denatured with SDS Few µg protein Optional reducing agents (e.g., DTT), boiling	∼1–2 kDa resolution for proteins	1D gel image with protein bands indicating molecular weight	High Multiple samples can be run simul-taneously	Simple and cost-effective High repro-ducibility Quantifiable Relatively fast (1–3 hours)	Requires standards or detection for MW Lower size limit approx. 5 kDa No spatial or structural info Not suitable for intact complexes
Mass spectrometry	Protein identity Amino acid sequence (tandem MS) Post-translation modifications	Ionizes molecules and separates them based on their mass-to-charge ratio (m/z)	Pure or complex biological samples (peptides, proteins) Minimal salt pg–ng range	High mass accuracy (up to 0.001 Da)	Mass spectra (m/z vs. intensity) peptide maps identification lists post-translational modifications	Medium to high Modern instru-ments can process dozens to hundreds of samples/day	Highly sensitive Precise molecular identification Suitable for complex mixtures Low sample concentration and volume required	Requires expensive equipment and expertise Sample preparation critical Quantification can be complex Reliable for smaller proteins (<40 kDa)
Western blot	Protein identity Degradation products Post-translation modifications	Detect specific proteins in a sample through antibody binding after gel electro-phoresis	Few µg protein Requires specific detection antibody	Moderate (depends on antibody specificity)	Bands on membrane Qualitative / semi quantitative with densitometry	Moderate	Highly specific Low cost Compatible with complex mixtures	Needs good antibodies No structural information Semi-quantitative only
nanoDSF	Thermal stability Aggregation onset	Label-free technique that measures protein stability → detecting changes in intrinsic tryptophan fluorescence during thermal unfolding	Few µL approx. 10 µL Works at low concentrations	Low – transitions tempera-ture curves unfolding curves	Unfolding curves Inflection points	High	Label-free Quick and easy Works in many buffers Low sample volume required	Tryptophan & tyrosine are needed in sequence No molecular weight information No shape / structure information
Mass photometry	Multi-merisation Aggregation Molecular weight	Label-free technique that measures the mass of single molecules in solution → detecting light scattering as they land on a glass surface	pM – nM range approx. 10 µL Protein size >40–50 kDa	2% mass accuracy for >40 kDa	Histogram of molecular mass distribution	Medium	Low sample concentration and volume required Fast method Label-free	Does not work with small proteins (<40–50 kDa) Sensitive to buffer composition
Dynamic light scat-tering (DLS)	Multi-merisation Aggregation Particle diameter Poly-dispersity index (PDI)	To determine the size distribution of particles or proteins in solution → measuring fluctuations in scattered light caused by their Brownian motion	Low volume Low concentration possible	Poor 20%–30%	Size distribution plots (intensity/volume)	High	Low sample volume required Quick and easy	Cannot resolve heterogenous mixtures No structural details Only hydrodynamic radius
Size exclusion chromatography (SEC)	Multi-merisation Aggregation Molecular weight	Separates molecules based on their size as they pass through a porous column matrix	Approx. 50 µg protein	Medium (operation-based resolution)	Elution profile	Medium	Good for quantifying multimers Often established in lab	Requires standards or detection for MW Diluted sample Only hydrodynamic radius
Size exclusion chromatography Multi angle Light scattering (SEC-MALS)	Molar mass Size Oligomeric state Aggregation	Molecules separated by SEC, then analysed by MALS detector	Approx. 100 µL 0.5–10 mg/mL protein	Moderate Limited by SEC resolution	Molar mass vs. elution volume Chromato-grams Radius of gyration (Rg)	Medium	Absolute molar mass Detects aggregates	Limited by SEC resolution Potential interactions with column matrix Dilution effects
Analytical ultracentrifugation (AUC)	Molecular weight Shape Heterogeneity	Sedimentation in strong centrifugal field	Approx. 400 µL 0.1–1 mg/mL protein Highly pure	High	Sedimentation coefficient distribution Molar mass distribution	Low 6-24 hours	No column artefacts High resolution	Long experimental time Expertise for data analysis Expensive instrumen-tation
Circular dichroism (CD)	Secondary structure Folding transitions	Secondary structure of proteins → measuring their differential absorption of left- and right-circularly polarized light	Approx. 0.2–1 mg/mL 200–500 µL	Low	Spectra 190–250 nm Melting temperature (Tmelt) curves	Medium	Ideal for folding studies Useful when intrinsic fluorescence is insufficient	Limited structure resolution Requires pure buffer Time consuming
Small-angle X-ray scattering (SAXS)	Size and shape Molecular weight Confirmational variability	Provides information about the size, shape, and structure of molecules in solution → measuring the scattering of X-rays at small angles	Soluble macro-molecules (proteins, complexes) Approx. 0.1–10 mg/mL Monodisperse preferred	Low	Scattering curves Pair distribution	Low	Structural information in solution Detects flexibility	Time consuming analysis Requires high-quality samples Expensive
Electron microscopy	Visualization of molecules Detection of aggregates	High-resolution imaging technique → uses a beam of electrons transmitted through a thin sample to visualize its ultra-structure at high resolution	Must be fixed and stained (e.g., with heavy metals)	Nanometer to a few Ångströms	High-resolution grayscale images of ultrastructure or particles	Low to medium Preparation and imaging are time-intensive (hours to days per sample)	Suitable for nanoscale morphology and particle size analysis	Expensive and complex Low throughput

Additionally, the table outlines the strengths and limitations of each technique, such as sensitivity, throughput, and applicability to complex samples, alongside with potential challenges, including resolution limits or dependency on specific protein properties. This balanced comparison is intended to guide future research and practical implementation.

## 5 Discussion

This study aimed to compare commonly used analytical methods for early-stage assessment of antibodies and antibody-derived constructs. Rather than proposing a single workflow, we evaluated how different orthogonal methods, based on distinct physiochemical principles, can be combined to assess key quality attributes such as purity, folding, stability, multimerization and aggregation status (Simon et al., 2023). By highlighting the strengths and limitation of each method, we provide a practical perspective on how to build a robust analytical strategy using accessible tools.

Our results demonstrated that the full-length antibodies Ab1 and Ab1-scFv1 maintain predominantly monomeric states and exhibit high thermal and structural stability, as confirmed by mass photometry, DLS, SEC, and nanoDSF analysis. The slight increase in hydrodynamic size and altered unfolding profiles upon scFv fusion highlight subtle conformational changes, while retaining favourable stability characteristics. Notably, mass photometry detected a low-abundance dimer population (approximately 2%) for Ab1-scFv1 that was not observed with other methods, highlighting its sensitivity in identifying minor oligomeric species. Conversely, the smaller scFv fragments, including bi-scFv2-scFv1, showed a propensity for multimerization and aggregation, as reflected by higher polydispersity indices in DLS and earlier elution peaks in SEC. This highlights that antibody fragments and fusion proteins require careful characterization, as they may behave differently from full-length antibodies, in terms of folding, stability, self-association, and solubility.

Structural analysis using SAXS offered crucial insights into the conformational properties of the proteins in solution. The data confirmed extended, flexible structures in full-length antibodies and supported the spatial integration of fused scFv domains, albeit with some conformational flexibility. While SAXS may not be suitable for routine analysis, it provides valuable insights into the solution conformations of engineered antibody formats and can help assess structural compatibility in fusion proteins. This is especially important when assessing novel constructs where traditional high-resolution methods may not be feasible.

A multi-method analytical strategy builds confidence in the quality of antibody-based constructs, supporting reliable applications in both preclinical and translational studies. For example, by comparing results across SDS-PAGE, DLS, SEC, and SAXS, protein homogeneity and subtle aggregation tendencies could be more accurately assessed than by a single method alone (Temel et al., 2016; Alhazmi and Albratty, 2023). CD is particularly useful when intrinsic fluorescence is insufficient or when probing structural integrity beyond thermal stability, such as secondary structure content or conformational changes missed by nanoDSF (Atsavapranee et al., 2021).

Despite their value, routine use of SAXS and CD may be constrained by accessibility and technical requirements. More accessible alternatives include thermal shift assays and DLS, which provide information on thermal stability as well as particle size and oligomeric state in solution. Although these techniques lack the resolution of SAXS or CD, they are highly suitable for early-stage screening and routine assessment in pre-clinical research and industrial settings.

For *in vivo* applications, specific biophysical and biochemical quality thresholds help guide the selection and optimization. Purity and homogeneity should be evident in well-defined SDS-PAGE and SEC profiles. Endotoxin levels must be controlled, with <0.1 EU/µg typically required for mouse studies (Malyala and Singh, 2008; Jeong et al., 2025; American Pharmaceutical Review, 2018; ichorbio, 2022). DLS should indicate low PDI, ideally <20%, values between 20% and 30% may be tolerable, but values >30% usually indicates problematic aggregation or heterogeneity (ichorbio, 2022). For antibody‐based applications, the minimal acceptable melting temperature of therapeutic antibodies is typically in the range of 45 °C to 50 °C, considering the physiological temperature of 37 °C (Stevens, 2011). CD and nanoDSF can reveal unfolding behaviour; cooperative unfolding transitions often indicate well-folded, stable proteins, while baseline drift or multiple unfolding events suggest structural instability or domain interactions. Poor signal recovery after thermal denaturation may indicate misfolding or aggregation, which could impact *in vivo* performance.

In addition to ensuring biochemical and structural quality, many of the characterized attributes, such as thermal stability, aggregation tendency, monodispersity, and folding efficiency, are directly relevant to *in vivo* performance. Aggregated or unstable proteins can trigger immune responses, reduce circulation half-life, and impair target binding. Therefore, robust analytical characterization not only improves reproducibility in research but also helps to de-risk candidates in preclinical and translational pipelines. By aligning *in vitro* quality assessments with *in vivo* expectations, this orthogonal approach facilitates the development of antibody-based therapeutics with improved safety, efficacy, and developability profiles.

When working with recombinant antibodies or fusion proteins intended for use in animal studies, several biosafety aspects must be considered to ensure safe and ethical handling. These include the biosafety classification of the expression system (e.g., Expi293), as certain host organisms may pose specific risks or require defined containment levels. It is also essential to verify protein purity and assess endotoxin levels, particularly using assays such as the LAL test, to avoid unintended immune activation *in vivo*. Good laboratory practices (GLP) must be followed to prevent microbial contamination or cross-contamination during expression, purification, and storage. Additionally, all *in vivo* experiments should comply with institutional and national regulations for animal welfare, including prior ethical approval and adherence to humane endpoints. These measures collectively help ensure both experimental reproducibility and animal safety in preclinical research involving recombinant biologics.

In summary, this study underscores the importance of integrating orthogonal analytical methods for comprehensive characterization of antibody-based therapeutics. Such an approach not only improves the reliability of structural and biophysical assessments but also supports rational construct design, risk mitigation during development, and informed decision-making in downstream applications.

Although this study does not cover functional downstream assays or pharmacokinetic behaviour, additional quality dimensions, including target binding in complex biological matrices, immunogenicity, and off-target effects, are essential considerations in the transitions from bench to *in vivo* studies.

Next steps may include applying this analytical strategy to novel therapeutic candidates and correlating key biophysiological features with biological efficacy or stability in *in vitro* functional assays as well as *in vivo* performance. Altogether, a comparative, multi-method approach enables more informed decision-making in the early development of antibody-based therapeutics.

## Data Availability

The original contributions presented in the study are included in the article/supplementary material, further inquiries can be directed to the corresponding author.
