# Supplementary material for: Enhancing therapeutic antibody profiling: orthogonal strategies for stability and quality assessment
Source: Front Pharmacol. 2025 Sep 26;16:1667210. doi: 10.3389/fphar.2025.1667210 (PMC12510943; doi:10.3389/fphar.2025.1667210)
Supplement: Supplementary file 1 [file Supplementaryfile1.docx]

**Supplementary Figures:**

**Enhancing therapeutic antibody profiling: orthogonal strategies for stability and quality assessment**

Nicole G. Metzendorf^1*^, Inga Petersen^1^ and Greta Hultqvist^1^

1 Department of Pharmacy, Husargatan 3, Box 580, 751 23, Uppsala University, Uppsala, Sweden

*Corresponding authors: Nicole.Metzendorf@uu.se


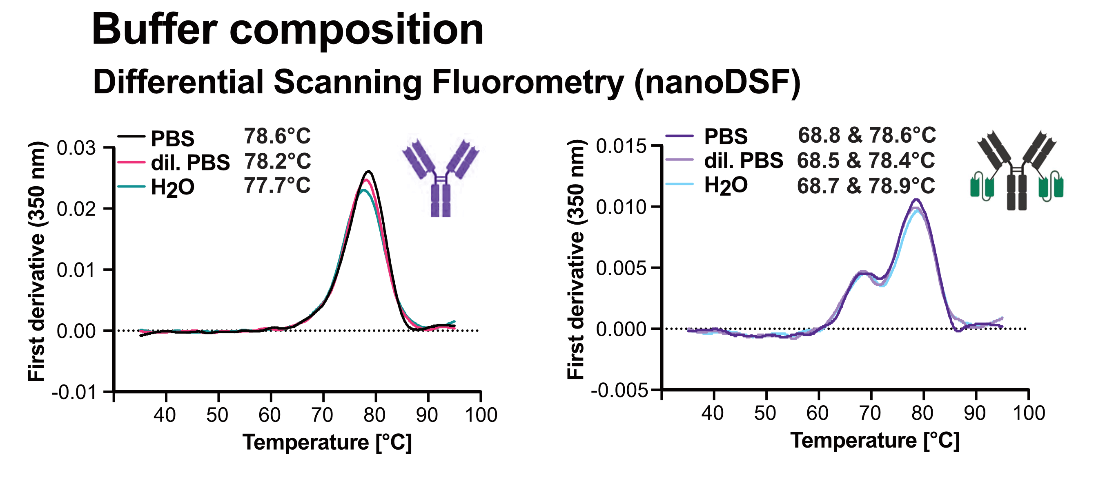


**Supplementary Figure S1. Effect of buffer composition on the thermal stability of Ab1 and Ab1-scFv1.**Thermal stability was evaluated using differential scanning fluorimetry (nanoDSF) under three buffer conditions: PBS, 1:5 (v/v) diluted PBS, and ultrapure water. Both proteins were analyzed at a concentration of 0.2 mg/mL (corresponding to 1.3 µM for Ab1 and 1.05 µM for Ab1-scFv1).


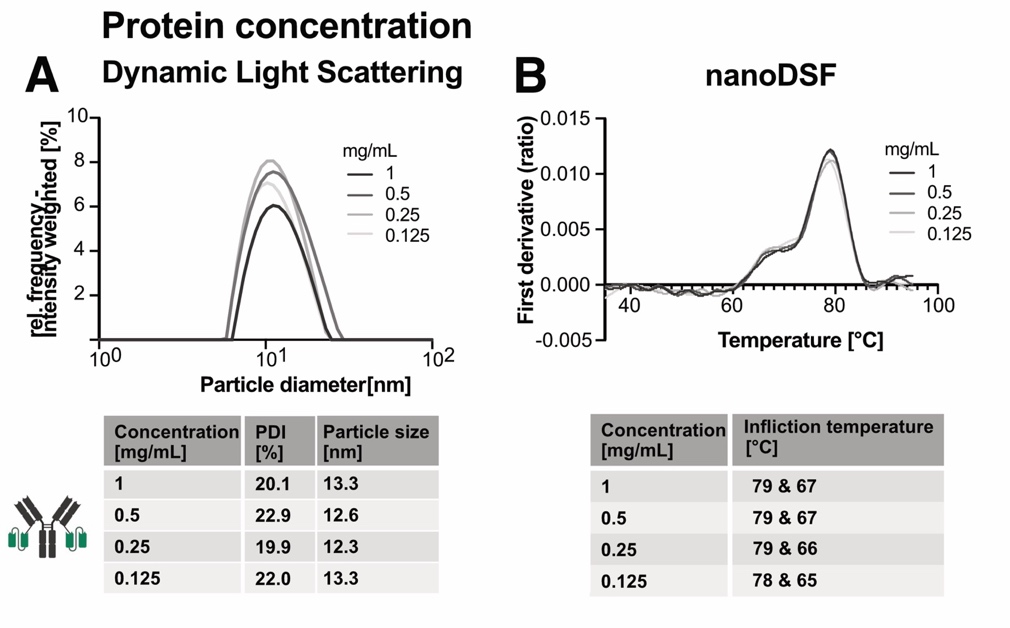


**Supplementary Figure S2. Effect of protein concentration on dynamic light scattering (DLS) and nano differential scanning fluorimetry (nanoDSF) measurements for Ab1-scFv1 (1 – 0.125 mg/mL).
(A)** Dynamic light scattering (DLS): Hydrodynamic diameter of Ab1-scFv1 measured at decreasing concentrations (1.0, 0.5, 0.25, and 0.125 mg/mL; corresponding to 6.67, 3.33, 1.67, and 0.83 µM, respectively), showing only a minor influence of concentration on particle size. Polydispersity index (PDI) values are provided in the accompanying table. **(B)** Nano differential scanning fluorimetry (nanoDSF): First derivative of the fluorescence ratio (350/330 nm) and corresponding inflection temperature (Tᵢ) at each concentration indicate no significant effect of protein concentration on thermal stability.


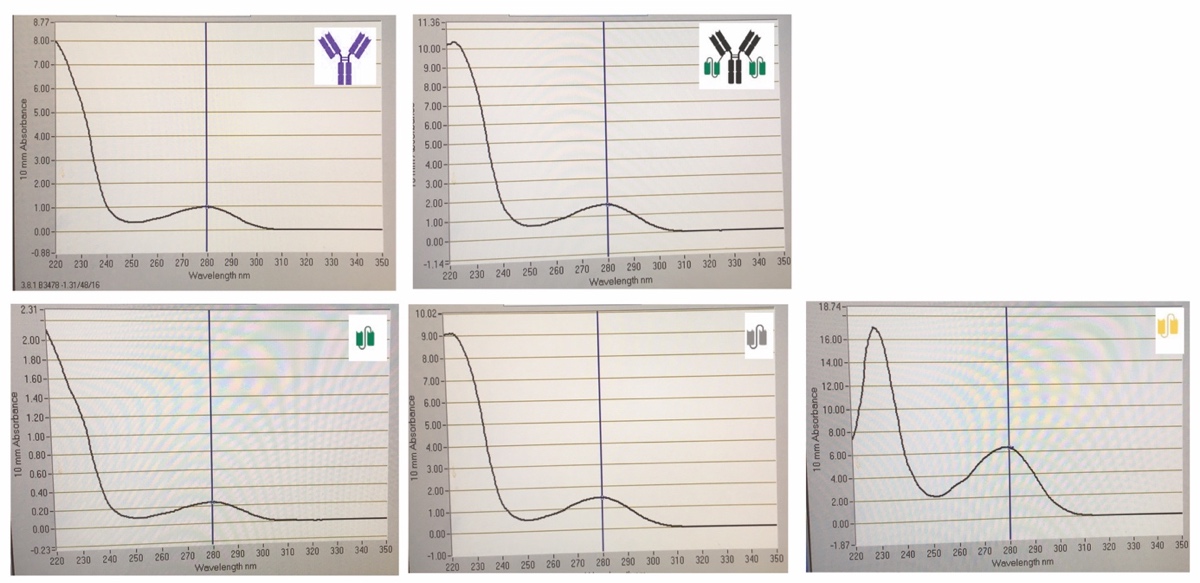


**Supplementary Figure S3. UV absorbance spectra of purified recombinant proteins measured by microvolume spectrophotometry (A₂₈₀).** UV absorbance spectra were recorded to assess the purity of each recombinant protein preparation. Aggregated proteins typically exhibit a shoulder around 220–230 nm due to increased light scattering, which can distort the standard absorbance profile. Schematic representations of each recombinant protein are included for reference.

**Supplementary Figure S4. SAXS quality assessment for Ab1, Ab1-scFv1, bi-scFv2-scFv2, and scFv1.** Small-angle X-ray scattering (SAXS) was performed to evaluate the solution-state structural properties and quality of Ab1, Ab1-scFv1, bi-scFv2-scFv2, and scFv1. Shown are representative plots used to assess SAXS data quality: pair-distance distribution functions P(r) (for Dmax), Guinier plots (for linearity and radius of gyration (Rg) determination), Kratky plots (to assess folding state and flexibility), and X^2^ values (to evaluate fit quality between experimental data and theoretical models). Rg reflects the average distance of scattering particles, indicating larger or more flexible molecules with a larger Rg and more compact or globular molecules with a smaller Rg. An IgG fusion protein ranges usually between >80 Å, whereas a scFv has approximately 15-30 Å. X^2^ (fit) represents the how well the model fits the data and values around 1 are considered a good fit.

Rg and X^2^ (fit) values are as follows: Ab1 – 76.76 Å and 1.0122, Ab1-scFv1 – 88.13 Å and 0.9904, bi-scFv2-scFv1 – 40.48 Å and 0.8584, and scFv1 – 31.97 Å and 1.070. Schematic representation of each recombinant protein are included for reference.
